# Effects of infertility on relationship quality and mental health outcomes in sub-Saharan Africa: A scoping review

**DOI:** 10.1371/journal.pgph.0005923

**Published:** 2026-11-10

**Authors:** Kenneth Juma, Cynthia Kairu, Merlyne Akwir Nungo, Merryline Chepkoech, Easter Olwanda, Sally Atieno Odunga, Ramatou Ouedraogo, Boniface Ayanbekongshie Ushie, Yohannes Dibaba Wado

**Affiliations:** 1 African Population and Health Research Center, Nairobi, Kenya; 2 Gender and Women’s Health, Nossal Institute for Global Health, School of Population and Global Health, University of Melbourne, Melbourne, Australia; 3 Kenya Medical Research Institute, Nairobi, Kenya; 4 School of Business and Economics, Vrije Universiteit Amsterdam, Amsterdam, The Netherlands; 5 Beshi King Development Services Limited, Abuja, Nigeria; University of Balamand Faculty of Health Sciences, LEBANON

## Abstract

Infertility is associated with significant psychosocial challenges, and relationship strain yet remains underprioritized in sub-Saharan African policy and research. We conducted a scoping review to examine the effects of infertility on relationship quality and mental health outcomes among people experiencing infertility in sub-Saharan Africa. We utilized the Joanna Briggs Institute methodology for scoping reviews and reported following the Preferred Reporting Items for Systematic Reviews and Meta-Analyses extension for Scoping Reviews. We searched databases including PubMed/MEDLINE, CINAHL, Web of Science, JSTOR, Scopus, Academic Search Complete, PsycINFO, and the Psychology and Behavioral Science Collection. Eligible articles focused on the effects of infertility on mental health and relationship quality in women, men, and couples experiencing infertility and were published between January 2000 and November 2025. We extracted data and conducted an iterative narrative synthesis. We included 70 articles: 36 quantitative, 5 mixed methods, and 29 qualitative. Four key themes emerged, which fit within the socio-ecological model: 1) infertility and relationship quality, 2) infertility and mental health, stigma and discrimination, 3) adaptive coping mechanisms, and 4) maladaptive coping mechanisms. Infertility was associated with anxiety, depression, and emotional distress. Studies revealed profound experiences of isolation and grief over unfulfilled reproductive goals, compounded by societal pressures. Diminished relationship quality was a common outcome among individuals with infertility, manifesting as intimate partner violence, verbal and emotional abuse, loss of intimacy, infidelity, and separation or divorce. Couples with infertility attempted multiple care pathways, including religious, biomedical, and traditional care. Lack of psychosocial support was common, and individuals often coped through substance and drug abuse, isolation, and active avoidance. Complexity of the relationship between infertility, mental health, and relationship quality, reinforces the need for urgent integrated individual and structural interventions that promote holistic fertility care and social support.

## Introduction

Infertility refers to the inability to achieve pregnancy after 12 months of unprotected sexual intercourse. It is classified as either primary infertility (where couples have never conceived) or secondary infertility (where couples who have previously conceived are unable to do so) [1]. Global estimates suggest that about 186 million people are affected by infertility, with the majority living in low-income countries [2]. According to a review by Cox et al. 2022, one in six individuals worldwide suffered infertility at some point in their lives [3]. This translates to a lifetime prevalence of 17.5% and a period prevalence of 12.6% as of 2022 [1]. A pooled estimate of lifelong infertility in Africa is 13.1%, while the pooled estimate of period infertility (the percentage of individuals or couples that are infertile at a particular time) is 16.4% [1]. In the sub-Saharan African (SSA) region, the prevalence of infertility ranges from 14.3% in The Gambia [4] to 21.2% in Ethiopia [5] and from a high of 30% in Nigeria [6] and 11.8% among women and 15.8% among men in Ghana [7]. While these estimates vary widely by study approach (hospital-based versus community-based) and were not drawn from nationally representative surveys, they offer crucial insights into the magnitude of infertility and the extent of data on the topic in SSA.

Compared to pregnancy, childbirth, and family planning, infertility remains underprioritized in sexual and reproductive health policy and programming despite its significant prevalence and varied consequences on people’s well-being [8]. Further, there is minimal attention to men, both in research and discussions around infertility [9]. Even so, the varying prevalences across settings emphasize the need for region-specific research and interventions that promote infertility-related policies for better access to infertility services and comprehensive support for people living with infertility [10].

Causes of infertility vary widely, with several myths and misconceptions around the causes (e.g., past contraceptive use, “past promiscuity”, bewitchment/witchcraft, and divine punishment, past abortion), particularly reflecting the social meaning ascribed to childlessness [11,12].

Infertility is associated with various health, social, and economic challenges. Couples often experience stress, anxiety, and depression, which can strain their relationship and is often associated with physical abuse [13]. Socially, they may not attend child-related activities or events, leading to feelings of loneliness and isolation, often worsened by cultural stigma [14]. Individuals and couples who experience infertility frequently face shame, anxiety, despair, ostracism, and low self-esteem, which can have negative consequences [8,9,15]. Infertility can lead to marital instability, resulting in separation and divorce [16,17]. Economically, infertility can impose significant financial burdens, often leading to catastrophic healthcare expenditures [18]. These costs may result in asset depletion, debt accumulation, and difficulty meeting essential needs [14,18]. The cost of infertility treatment, particularly assisted reproductive technology (ART), is often cited as a significant barrier to access, especially in low- and middle-income countries [19,20].

Notably, the high prevalence of infertility has not translated to increased availability and access to infertility treatment in Africa [20]. Access remains acutely limited, mainly due to the high cost of treatment, lack of appropriate infrastructure and skilled personnel, and the absence of public funding for medical services and medication [21]. A recent multiyear analysis showed that women who used fertility treatments had a lower risk of divorce up to 20 years after the marriage, whether the treatment succeeded or not [22]. The burden of seeking fertility treatment disproportionately falls on women whose social and cultural identity is defined by motherhood [23].

Existing studies in sub-Saharan Africa have examined aspects of the psychosocial consequences of infertility; however, there are no reviews that synthesized the evidence on the relationship between infertility and both mental health and relationship quality among men, women, and couples experiencing infertility. The lack of extensive research and documentation on the effects of infertility inhibits its prioritization as a policy-relevant topic needing urgent interventions [24]. Recent scoping reviews in Africa have focused on the fear of infertility [25], interventions addressing infertility-related gendered stigmatization [26], and the psychosocial aspects of infertility [27]. One scoping review, concluded in 2022 and published in 2024, focused on the effects of infertility on mental health in Africa [28]. This review aimed to close this gap by examining the effects of infertility on both mental health and relationship quality among women and men (including couples) experiencing infertility (whether primary or secondary) and community members in SSA. The review findings are crucial for highlighting gaps in fertility care and guiding targeted interventions toward holistic infertility care as part of family planning services.

We situate this scoping review within Bronfenbrenner's socio-ecological model (SEM) [29], which links individual, relational, community, and structural influences on social phenomena. The SEM is a useful framework for understanding how infertility affects relationship quality and mental health by situating individual experiences within layered social and environmental contexts [29]. The SEM organizes determinants and outcomes across five nested levels: (1) individual (e.g., personal psychological responses such as anxiety, grief, depression, and self-stigma); (2) interpersonal (e.g., impacts on marital communication, intimacy, and stability); (3) organizational/community (e.g., social stigma from extended family, religious institutions, or healthcare providers); (4) societal (e.g., pronatalist cultural norms and gender-role expectations, social pressures); and (5)] policy (e.g., lack of public funding and infrastructure for infertility care) [30]. This multi-level framework is especially relevant for SSA, where strong pronatalist cultures and structural barriers amplify the psychological burdens of infertility. This helps explain why the same condition can have different psychological and relational impacts across contexts, especially in settings where fertility is highly valued.

### Overarching review question

What is the extent of research conducted on infertility and its effects on mental health and relationship quality in SSA between 2000 and November 2025?

### Purpose of the scoping review

This review aimed to identify and synthesize the effects of infertility on mental health and relationship quality among individuals (women, men, and couples) experiencing infertility in SSA**.** Specifically;

1. To identify the scope and range of studies available on infertility, mental health, and relationship quality among individuals living with infertility in SSA
2. To map key findings from the studies on the effects of infertility on mental health and relationship quality among individuals living with infertility
3. To highlight gaps in the current evidence base in relation to settings, participants involved, study designs, and specific care and outcomes around infertility.

## Methods

This scoping review was conducted in accordance with the Joanna Briggs Institute (JBI) methodology for scoping reviews [31,32]. We followed five steps: *1)* defining the research question, *2)* identifying articles, *3)* selecting articles, *4)* extracting data and compiling, and *5)* summarizing and presenting the review findings in line with the Preferred Reporting Items for Systematic Reviews and Meta-Analyses extension for scoping reviews (PRISMA-ScR) [33]. We registered the scoping review protocol in the Open Science Framework (https://doi.org/10.17605/OSF.IO/5AX6F).

### Criteria for considering studies for this review

#### Type of studies.

We included all studies that addressed the relationship between infertility and mental health and/or relationship quality. We included primary quantitative studies of all designs: observational studies (cross-sectional, case-control, and cohort) and experimental designs (randomized controlled trials and quasi-randomized controlled trials). We also included primary qualitative studies using qualitative designs (e.g., ethnography, phenomenology, case study, grounded theory, and qualitative process evaluation). We included studies that used qualitative data collection methods (e.g., interviews, focus group discussions, key informant interviews, and observations) and analysis methods (e.g., thematic analysis, grounded theory, and framework analysis). We included mixed‐methods studies from which we could extract primary qualitative and primary quantitative findings.

We excluded any studies that were not primary quantitative or qualitative studies, including review studies (such as systematic, scoping and narrative reviews), commentaries or short communications, conference proceedings or workshop summaries, dissertations or theses, records for which full texts were unavailable, and studies or documents that do not include any aspect of infertility lived experiences around mental health and relationship quality among those living with primary or secondary infertility (Table 1).

**Table 1:** Inclusion and exclusion criteria.

Inclusion	Exclusion
**P = TARGET POPULATION**	
Women, men and couples experiencing infertility	Women not experiencing primary or secondary infertility, including other stakeholders not involved in the experiences of those with infertility
**C = CONCEPT**	
Address the relationship between infertility (primary or secondary infertility) and its relationship or effects on mental health. We will include studies exploring the relationship between infertility and marital and family relationships, including the coping mechanisms linked to these mental health and relationship quality outcomes.	Unrelated to infertility and its relationship with mental health or relationship quality. Studies addressing other aspects of infertility such as (e.g., prevalence, etiology or epidemiological studies), or those describing care processes and interventions such as assisted reproductive technology and surrogacy
**C = CONTEXT**	
Peer-reviewed literature reporting studies conducted in sub-Saharan Africa	Studies reporting findings from outside of sub-Saharan Africa
**TYPES OF EVIDENCE SOURCES**	
Literature available in any language; Full text available; Primary peer-reviewed research publication Qualitative studies using qualitative methods for data collection and qualitative data analysis approaches Quantitative studies using any study designs (cross-sectional, cohort, case-control and randomized controlled trials We also included mixed-methods studies where we could extract either quantitative and/or qualitative data	Reports, letters, editorials, commentaries, reviews, study protocols, book chapters, dissertations, short communications, posters, conference abstracts and records without full texts

#### Topic of interests.

The phenomenon of interest in this review was infertility (whether primary or secondary infertility) and its relationship or effects on mental health outcomes (i.e., the range of psychosocial challenges such as anxiety, depression, low self-esteem, and suicidal Ideation). We focus on the relationship between infertility and marital relationships (i.e., divorce, separation, infidelity, sexual satisfaction, and forms of gender-based violence). We also explored the coping mechanisms associated with these mental health and relationship quality outcomes, both adaptive and maladaptive coping mechanisms. Finally, we also explored the supportive structures and systems available to individuals experiencing infertility.

#### Type of participants.

We included studies that presented the perspectives of sexually active men and women (including couples) experiencing infertility, regardless of whether they had primary or secondary infertility. We also included studies that considered the perspectives of family members, in-laws, and community members.

#### Setting or context.

We included studies conducted within countries in SSA. Study settings included formal and informal health care settings, communities (e.g., churches), and informal settings (e.g., traditional birth places, shrines).

### Search methods for the identification of studies

#### Data sources and search strategy.

The search strategy incorporated keywords, Boolean logical operators and their respective synonyms, truncations, adjacency searches and inverted commas. The search strategy followed three distinct stages. First, we conducted an electronic search of the MEDLINE/PubMed, CINAHL, Web of Science, JSTOR, Scopus, Academic Search Complete, PsycInfo, and Psychology and Behavioural Sciences Collection databases in June 2024 and updated it in November 2025. The following words made up the variants of search terms across the targeted databases: “Infertility OR childless* OR sterile OR impoten* OR infecundity OR “primary infertility” OR infertile OR “secondary infertility” OR “Assisted Reproduction” OR “In Vitro Fertilization” OR “Fertility Clinics” AND “Intimate partner violence” OR IPV OR “marital dissolution” OR divorce OR separation OR “domestic violence” OR “domestic abuse” OR anxiety OR depression OR stigma OR stress AND “sub-Saharan Africa” OR “East Africa” OR “Southern Africa” OR “West Africa” OR “Central Africa.” We searched each database using a specific search strategy, as detailed in S1 Table. Second, we screened the reference lists of all eligible studies to identify additional eligible articles.

#### Screening of studies.

We used Rayyan software, an online tool that allows reviewers to independently screen several articles simultaneously. We uploaded search results to Rayyan for de-duplication and to manage the selection process. At least two review authors screened article titles and abstracts independently to determine eligibility according to the pre-agreed inclusion and exclusion criteria. For articles lacking an abstract, we reviewed the full text. At least two review authors independently conducted full-text screening and documented the reasons for exclusion. The review authors resolved disagreements through discussion or, when required, by involving a third review author. Where appropriate, we contacted the study authors for further information. Where the same study, using the same sample and methods, was presented in different reports, we collated these reports so that each study (rather than each report) was the unit of interest in our review. We included a PRISMA flow diagram to show our search results and the process of screening and selecting studies for inclusion.

#### Data extraction.

Three independent reviewers (EO, MC, MA) extracted data from each article. We used a pre-designed data extraction template in Microsoft Excel to extract general study details (including study title, publication year, author, country, study type, study design, data collection tools, study objectives, setting, sample size, participants’ sex, age, religion, education, marital status, type of marriage, chronology of marriage, type of infertility, and duration of infertility (years)). We also extracted specific details covering infertility-related themes (such as psychosocial challenges/problems due to infertility (stress, forgetfulness, lack of sleep, loss of appetite, poor concentration, anxiety, depression, anger, low self-esteem, hopelessness, and suicidal ideation). We also extracted information on the effects of infertility on marital and family relationships (divorce, separation, infidelity, reduced sexual satisfaction, intimate partner violence, and verbal abuse, emotional abuse). We also included details on adaptive coping and maladaptive coping mechanisms, and support systems. We also gathered information on study limitations.

The data extraction tool was developed according to Pollock et al.’s guidance on best practices for data extraction, analysis, and presentation within scoping reviews [32]. We piloted the form across a sample of five studies. Based on the pilot results, we revised the data extraction form as needed.

#### Data synthesis.

We conducted an iterative narrative synthesis to analyze and integrate key findings from the review, as described by Popay et al. [34]. First, we inductively analyzed the qualitative data to identify emerging themes. Two authors independently coded the extracted qualitative data using NVivo 12 and a codebook that was developed and iteratively refined as the analysis progressed. The coded data were then organized into themes and subthemes. To integrate the qualitative and quantitative findings, the quantitative findings were mapped onto the existing themes and subthemes from the qualitative findings, and additional themes were created where the existing themes did not fit.

#### Assessment of methodological quality.

An assessment of methodological quality was not conducted, which is consistent with accepted scoping review methods [35].

## Results

Our search strategy identified 3013 articles. After excluding 228 duplicates, we were left with 2,785 articles for the title and abstract screening. After this screening and eligibility assessment, we excluded 2,705 articles outside the scope of this review, leaving 80 articles for full-text screening. We excluded an additional 10 articles during full-text review for reporting the wrong outcome and included the remaining 70 articles in the scoping review (Fig 1).

**Fig 1:**
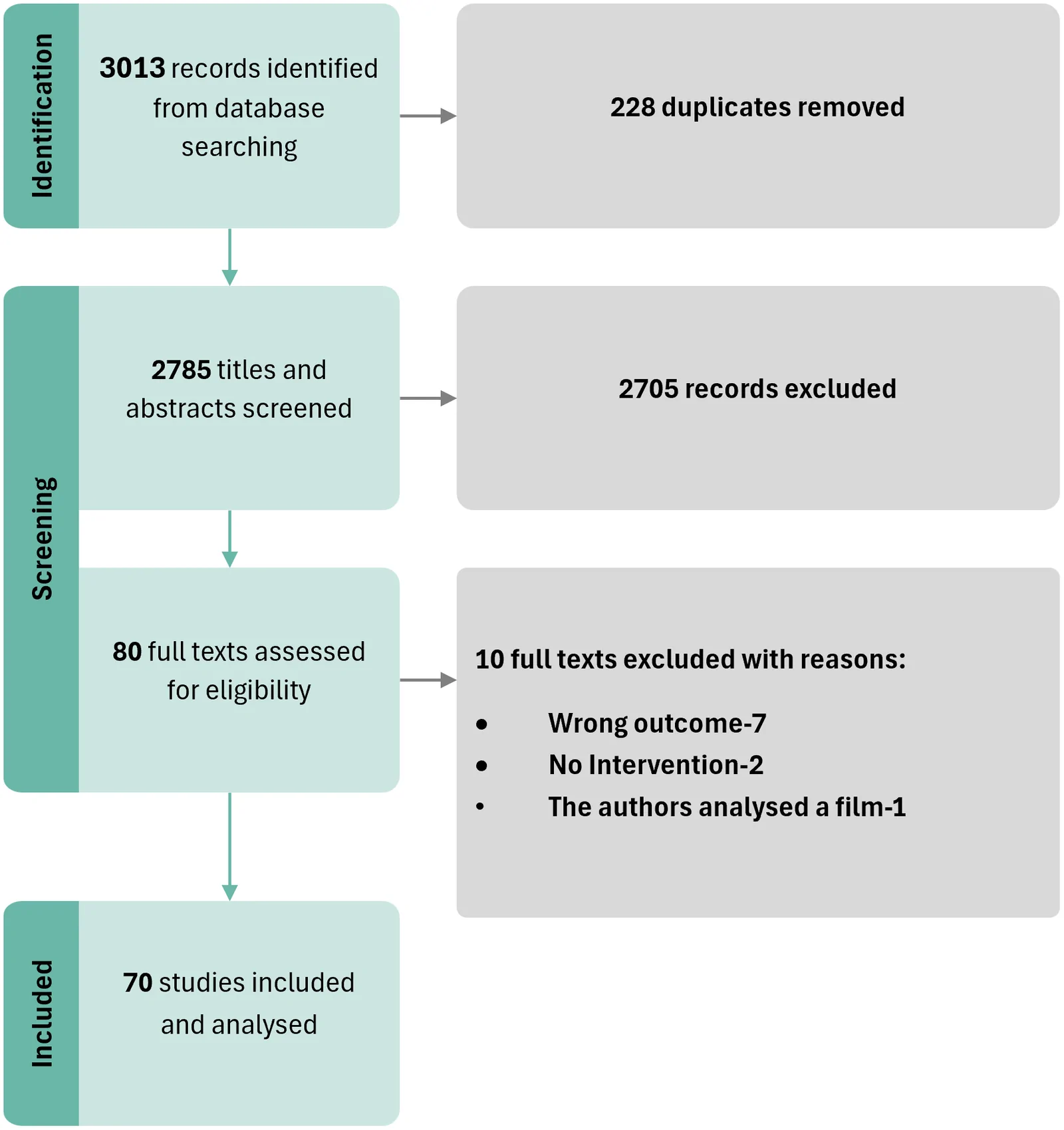
PRISMA flow diagram of the literature search and selection process.

### Study characteristics and geographic distribution

The highest proportion of the 70 articles included in this scoping review were published from Nigeria (35.7%) and Ghana (27.1%). Other countries represented included Ethiopia (8.6%), South Africa (4.3%), Malawi (4.3%), Zimbabwe (4.3%), Gambia (2.9%), and the remaining countries had 1.4% each (Zambia, Tanzania, Uganda, Senegal, Rwanda, Mali, and Kenya). Two multi-country studies were included: the first covered Tanzania and Ghana, while the second covered Cameroon, Gabon, Lesotho, Liberia, and Sierra Leone, at 1.4% each (Fig 2).

**Fig 2:**
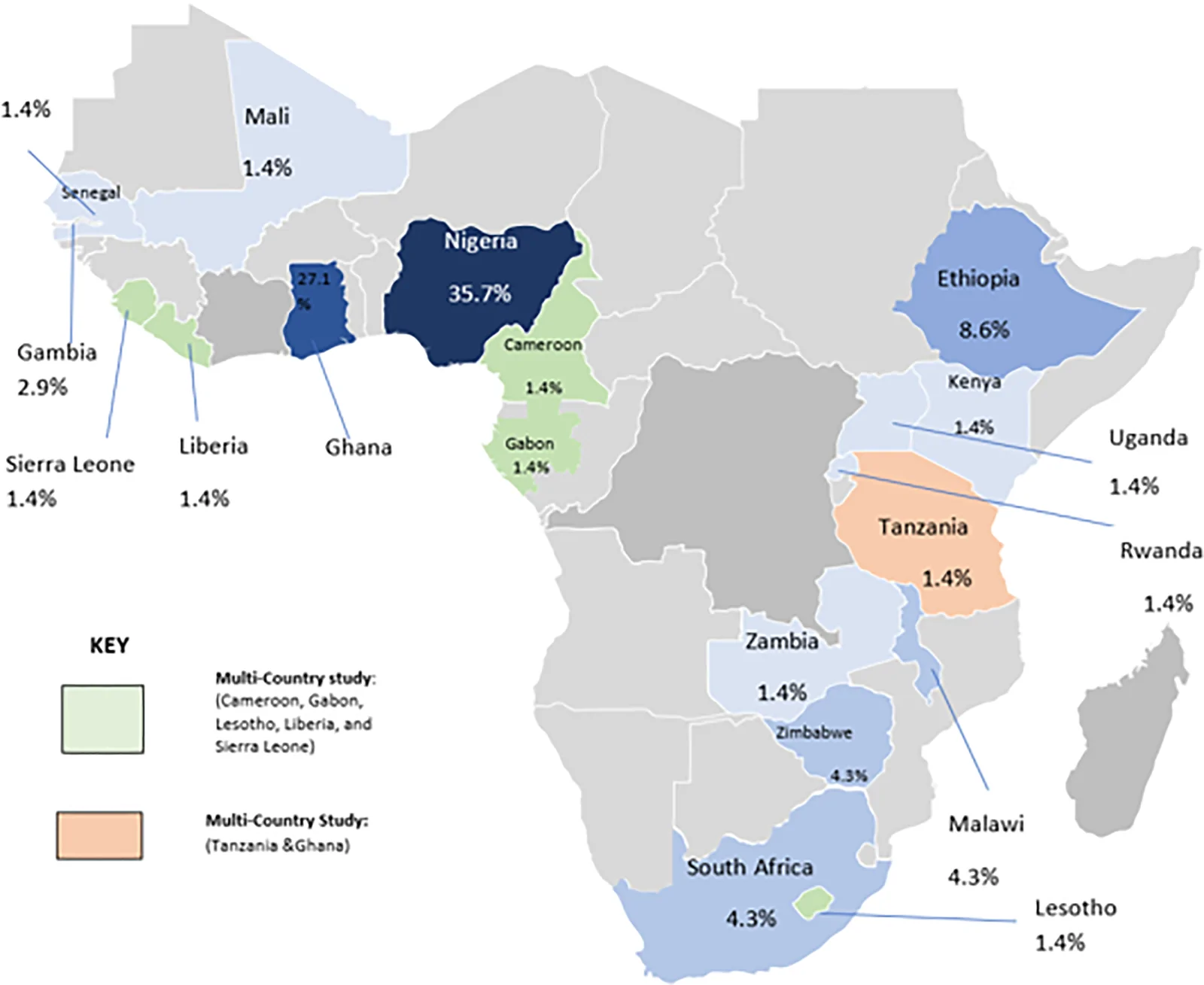
Density map of the proportion of published articles by countries. Map generated using an openly licensed base map from OpenStreetMap contributors (CC BY 4.0). Administrative boundaries and study locations were highlighted by the authors. The figure illustrates the geographic distribution of the study sites included in the analysis.

Most of the articles were published between 2010 and 2019 (31.1%), followed by those from January 2020 to December 2024 (35.7%), with smaller fractions published between 2000 and 2009 (14.3%) and in 2025 (12.9%). Most articles published data focused on women (65.5%), with about one-quarter reporting data from both sexes (24.1%), and those focused exclusively on men accounted for just 10.3%.

Most articles used data collected within health facility settings (74.3%), with community settings at 18.6%, Medical colleges and Traditional Health Practitioners (THPs) at 1.4% each, and other contexts at 1.4%. Lastly, 2.9% of the studies included did not report the setting in which they were conducted. Slightly over half of the articles were quantitative studies (55.2%), followed by qualitative studies (39.7%) and mixed-method studies (5.2%). Table 2 presents a detailed description of the included articles.

**Table 2:** Characteristics of the included articles.

Characteristics		Number	%
**Country**	Nigeria	25	35.7%
	Ghana	19	27.1%
	Ethiopia	6	8.6%
	South Africa	3	4.3%
	Malawi	3	4.3%
	Zimbabwe	3	4.3%
	Gambia	2	2.9%
	Zambia	1	1.4%
	Tanzania & Ghana	1	1.4%
	Senegal	1	1.4%
	Rwanda	1	1.4%
	Mali	1	1.4%
	Kenya	1	1.4%
	Cameroon, Gabon, Lesotho, Liberia, and Sierra Leone	1	1.4%
	Tanzania	1	1.4%
	Uganda	1	1.4%
**Publication Year**	2000-2009	10	14.3%
	2010-2019	26	37.1%
	2020-2024	25	35.7%
	2025	9	12.9%
**Sex**	Female	46	65.7%
	Both	17	24.3%
	Male	7	10.0%
**Study setting**	Healthcare	52	74.3%
	Community	13	18.6%
	Not reported	2	2.9%
	Other	1	1.4%
	Medical College	1	1.4%
	Traditional Health Practitioners (THPs)	1	1.4%
**Study Type**	Quantitative	35	51.5%
	Qualitative	28	41.2%
	Mixed-Method	5	7.4%

### Infertility and relationship quality

Fifty-one studies reported on the relationship between infertility and relationship quality. Findings showed an association between infertility and poorer relationship quality that co-occurred with infidelity, separation and divorce, decreased sexual satisfaction, and physical and emotional abuse. Additional details on the findings from each study are presented in S2 Table.

#### Reduced sexual satisfaction.

Four studies in Ghana reported on the relationship between infertility and reduced sexual satisfaction [38,39,42,45]. Infertility severely affected the sexual pleasure and lives of individuals and was associated with poor sexual relationships, sexual disengagement, and reduced sexual satisfaction, with some characterizing the sexual activity as “pointless”, “fruitless”, “unfulfilling” and “unenjoyable” [36–38]. Studies across SSA confirmed this notion, and specific examples in Ghana highlighted that 61.6% (77/112) of couples experiencing infertility had sex solely for reproduction, and 64.3% (72/112) experienced unfulfilling and unenjoyable sex [39]. Findings also emphasized the social meaning of infertility through the substantial pressure exerted on individuals living with infertility, ultimately affecting their sexual pleasure and satisfaction [37]. Other studies demonstrated the intimate link between infertility-related stress and controlling behavior (such as coercive sexual behavior), emotional strain (mainly from accusations of abortion and conflicts over communication), and erosion of intimacy and social functioning in marriage or relationships [40,41]. Qualitative studies also affirmed how this distressed marital environment contributed to reduced sexual interest due to its perceived futility [38]. One man recounted how, for six months, he had not engaged in sexual activity with his wife because it is a waste of energy: “Why do you continue to have fruitless sex with your wife” [42].

#### Infidelity.

Nine studies from Cameroon, Ghana, Malawi, Nigeria, Rwanda and Zimbabwe reported on the association between infertility and the occurrence of extramarital affairs or infidelity. Studies emphasized the association between infertility and infidelity, with one study in Rwanda asserting that more men experiencing infertility (16%; 41/254 versus 3%; 6/189) were engaged in extramarital relationships compared to women [43]. Findings from a multi-country analysis confirmed that married women experiencing infertility were two times more likely to engage in marital infidelity compared to married women with children [44]. Qualitative studies in Zimbabwe, Nigeria, and Rwanda described a pattern of extramarital affairs by both men and women whose partners were experiencing infertility, ostensibly to save their marriages [6,43,45,46]. These extramarital affairs bred doubt within the relationship, weakening trust and stability. One respondent in Zimbabwe lamented how infertile men engage in multiple extra-marital relationships, hoping to impregnate one woman along the way, and this practice exposes the couple to risks of sexually transmitted infections and HIV [46]. In some cases, the emergence of infidelity was due to pressure from in-laws, with a male respondent in Nigeria acknowledged that “the husband is often urged to take another wife” [47]. A study in Malawi reported that it was the husband pushing the wife to engage in an extramarital affair, by suggesting to “hire a man to impregnate her”, acknowledging his own inability to achieve a pregnancy [48]. Not all extramarital affairs ended well, and some women in Ghana narrated how they endured painful rituals as punishment after their secret extramarital affairs were discovered [37,42].

#### Physical and emotional abuse.

Fifteen studies highlighted how infertility intersects with various forms of violence, reshaping the lives of women and men far beyond their private spheres [43,49–51]. Violence was perpetrated not only by husbands or partners but also by other family members [49,52]. A Gambian study found that women facing infertility were beaten by husbands and female in‑laws for supposedly not being “real women” because they could not conceive [52]. Quantitative studies from Ghana, Nigeria, South Africa, Tanzania and Zambia show that around 38%–64% of women experiencing infertility face violence [11,51–54]. The most common forms were psychological and verbal abuse, though many women endured multiple, overlapping forms of violence, including physical assault, sexual violence, emotional aggression, and deprivation [51,55].

Qualitative findings revealed how infertility permeated women’s and men’s lives across social, professional, and intimate spheres [41]. Women living with infertility were subtly dismissed at the workplace, with assumptions that they lacked caregiving responsibilities, reinforcing the belief that womanhood is incomplete without motherhood, and this caused deep emotional hurt [41]. Women faced ridicule and degrading comments that intensified feelings of inadequacy and isolation [46]. Some women in The Gambia, Senegal, and Malawi recounted how people in the community called them “witches” and labeled them “barren” [40,56,57], while other women lamented such demeaning remarks from their own husbands/partners, with one mentioning how her husband insulted her and called her names like “you dog, you are very stupid” [48]. Such verbal abuses strained relationships and escalated to intimate partner violence, with infertility becoming the fault line that exposed rigid gender expectations and pressures within families and communities [58]. Few studies also revealed how men experienced profound but often hidden suffering by enduring humiliation, public ridicule, and jokes questioning their masculinity. In Zimbabwe, men in these situations felt compelled to hide their distress, protect their partners, and silently carry shame and emotional pain [46].

#### Separation/divorce.

Twenty-three studies in this review showed that infertility was associated with increased marital tension and mistrust, and most men and families often blamed women whenever couples were childless [59]. Family members and in-laws exerted pressure on infertile couples, sometimes leading to separation and dissolution of marital unions, and several women and men living with infertility had multiple failed relationships and divorces [60]. Quantitative studies found that many women (e.g., Nigeria - 61.7%; 127/205) observed changes in their partner’s behaviour, relationship dynamics, and stability, and nearly half (Nigeria - 54.2%; 111/205) experienced increased marital discord [61]. Across contexts, infertility was associated with higher risks of relationship breakdown and divorce—for example, up to 95% (8319/8757) of childless women in Ethiopia experienced divorce within 20 years, with 85% (7444/8757) divorcing in the first five years—illustrating how infertility rapidly and severely undermines marital stability [17]. Men often resorted to secret arrangements and explored alternative childbearing plans to achieve pregnancy, as was the case in South Africa, where some men resorted to polygamy, leading to separation [62,63].

Qualitative studies described experiences of infertility in the context of relationship challenges, including conflict, instability, and marital breakdown. In Malawi, some men responded by seeking second marriages or turning to close female relatives—such as a wife’s sister—for informal surrogacy [57]. Across many African communities, the return of the bride price when a woman does not conceive further weakened marital bonds, effectively dissolving kinship ties and releasing both partners to pursue their reproductive goals independently [57].

### Infertility and mental health

A total of fifty-three studies highlighted the relationship between infertility and the occurrence of poor mental and psychological outcomes across multiple African contexts. Quantitative studies from Nigeria and Ethiopia reported high prevalence of depressive symptoms among individuals experiencing infertility (17–39% in Nigeria and 42.1% in Ethiopia), with varying levels of severity ranging from mild to severe depression [64–66]. Across studies, infertility was associated with emotional distress, anxiety, mood disturbances, and reduced psychological functioning, suggesting that mental health challenges are a pervasive consequence of infertility experiences. Evidence also indicates that psychological distress may intensify with prolonged infertility and repeated treatment failure [67].

Findings from qualitative studies further elucidate the understanding of these experiences by showing that infertility-related distress is shaped by uncertainty, fear of the future, and perceived or actual social expectations [67]. The studies show that participants commonly described persistent worry, anxiety, sadness, and emotional exhaustion from persistent concerns about their fertility status and multiple unsuccessful attempts to find a resolution [38,67]. In some instances, infertility was associated with severe psychological outcomes, including suicidal ideation, particularly among men [68].

We also identified the social dimensions of psychological distress associated with infertility. Feelings of loneliness, isolation, and social disconnection emerged across studies, with participants reporting withdrawal from family and peers due to stigma and societal pressure surrounding childbearing [69]. Social scrutiny, gossip, and expectations to conceive shortly after marriage further exacerbated anxiety, rumination, and sleep disturbances, while experiences of dehumanization often culminate in diminished self-worth and intensify emotional suffering [38].

Furthermore, two studies reported that infertility-related distress affects daily functioning and cognitive well-being. Individuals experiencing infertility experience difficulties with concentration, persistent intrusive thoughts, and reduced ability to manage routine activities and self-care [69,70]. Feelings of desperation and helplessness were reported among those who had multiple attempts at medical investigations and unsuccessful fertility treatments [6].

A more pertinent manifestation of societal reception to infertility was the presence of pervasive stigma (enacted) – represented by negative attitudes and behaviors directed towards persons experiencing infertility and was associated with social exclusion and public humiliation [46]. Studies across SSA presented accounts of how women and men with infertility were judged, mocked, gossiped about, labelled (e.g., witches or barren), and faced contemptuous ridicule from partners, family, and community members, and in all settings – workplace, religious, and family settings [41]. Infertility stigma was mainly driven and reinforced by various factors including limited biomedical understanding of infertility, societal framing of infertility as a curse [11,41], infertility as a personal failure to achieve parenthood [8,71], and blame of infertility being a result of past transgressions [41]. Some of these stigmatizing expressions emanated from comparisons of women experiencing infertility with their peers, neighbors, and in-laws, as narrated by a woman in Ghana who said, “I got married before a neighbor, but she gave birth almost immediately [72]. Everyone is talking about me because I have kept long [in giving birth” [72]. Qualitative studies also highlighted how men faced such demeaning experiences from close relatives and neighbors who keep their children away from people who are childless. One man in Ghana recalled how he was humiliated and insulted for sending a neighbor’s child to the shop, and the mother [of the child sent to the shop] told the man, “to impregnate your wife and have your own children whom you can send on errands” [46].

Feelings of internalized stigma and self‑blame were also frequent among individuals experiencing infertility. Individuals often absorbed these societal beliefs that equated fertility with personal worth, and this contributed to social isolation and hopelessness among affected persons, intensifying feelings of guilt and avoidance of care, self-questioning, and sadness when comparing themselves to those with children [41]. Other women blamed God, and as one woman remarked in Ghana, “life is not fair; I sometimes feel God shouldn’t have created me” [72].

This internal distress compounded experiences and manifestations of stigma and was associated with widespread social exclusion. Across studies in Ghana and Nigeria, individuals living with infertility were frequently marginalized within their communities, excluded from social gatherings, and denied participation in events centered around children. Quantitative findings consistently reinforced this pattern of social exclusion [69,73]. Beyond everyday social interactions, infertility also exposes individuals to cultural and ritual-based discrimination. Ethnographic accounts describe exclusionary burial practices for those who experienced infertility, signaling a stigma that extends beyond life. In some places, ritual purification practices have involved degrading and violent acts, such as stripping women naked and using thorns to scour their genitals—practices justified through cultural narratives linking infertility to moral transgression or impurity [42].

### Coping mechanisms

Six studies from Ethiopia, Ghana, Kenya and Nigeria reported on the various adaptive and maladaptive mechanisms that individuals living with infertility employ to cope with the experience of infertility and its social consequences (e.g., stigma, marital strain, uncertainty about the future). None of the studies in this review focused explicitly on any coping responses directed at mental health symptoms, but rather at the stressors generated by infertility — such as social pressure, repeated treatment failure, and fear of relationship dissolution.

#### Adaptive coping mechanisms.

There was a rich interplay between infertility and seeking spiritual, traditional, and biomedical interventions. Four studies in Zambia, Ghana, Ethiopia and Mozambique reported reliance on spiritual coping, and in some cases, positive religious coping was significantly associated with higher levels of somatization and anxiety, whereas negative religious coping was significant and positively correlated with age, duration of infertility and depression [67,70,74,75]. Individuals in these studies articulated beliefs in divine timing and the power of prayer, conveying hope grounded in their spirituality [41,72,76].

Across multiple settings, studies show a strong reliance on traditional (herbal) and faith‑based healing before or alongside biomedical care. In Mali, 71% (37/52) of participants used both traditional “African medicine” and Western treatments, often concurrently [77]. Similar patterns appear in Nigeria-57% (237/415) and Zimbabwe-80% (249/311), where the majority of individuals first sought help from traditional or faith‑based healers before turning to biomedical options. Together, these studies highlight the widespread use of pluralistic health‑seeking practices in navigating infertility [78,79].

Regarding biomedical treatment approaches, a hospital-based study in Nigeria showed that a significant portion of the population used medical treatments (62%; 257/415), while surgical interventions were utilized by about (33%; 137/415) of individuals [79]. The financial burden of these treatments was evident, with many participants expressing that the cost was secondary to the value of having children, emphasizing the profound emotional and social implications of infertility [47]. There has been an increasing demand for infertility treatment in SSA, particularly the demand for assisted reproductive technologies (ART), yet access to quality ART remains acutely limited. In many countries, ART services are very costly and primarily delivered by the private sector [41], except in Nigeria and South Africa [80]. Whittaker et al. (2023) observed that in SSA, Kenya had the fewest fertility clinics at 11, followed by Ghana at 18, South Africa at 40 and Nigeria with the highest number at 96, primarily set up to attend to international clients [21]. With the exceptions of Uganda and South Africa, where private insurance compensates ART treatments, and Tanzania, where the government provides partial coverage, other SSA governments barely prioritize ART in their policies. Tabong and Adogo (2013) reported that some women accepted their situation and resorted to pursue other things, including education, after waiting for several years to become pregnant in vain [42].

Mashaah et al. reported a spectrum of coping strategies, including acceptance and not pursuing serious relationships, choosing to focus on enjoying life, and avoiding the stress and stigma of remarriage or being labeled barren [81]. This finding aligns with Behude et al., who report that individuals facing infertility may come to terms with the possibility of not having biological children [67]. This shift in mindset helps them reframe their situation, enabling a focus on moving forward, pursuing alternative routes to parenthood, or embracing a life without children [67]. In contrast, other individuals opt for commitment to family-building through child fostering and adoption [67,81]. Likewise, Musa et al. reported that more than half (59.2%) of the study participants considered adopting a child as a coping strategy in Nigeria [61].

Anabila Adda et al., Behude et al., and Araya et al. reported spousal support as a key coping strategy, recognizing and valuing spouse’s persistent efforts despite repeated conflicts. These studies described situations where separation seemed possible, yet the couple chose to stay married, providing the emotional support needed to navigate relationship stress [38,67,74]. Likewise, Musa et al. reported that involving partners and getting financial aid were coping strategies for most individuals in Nigeria [61]. The scoping review findings illustrated that the adaptive coping strategies were not mutually exclusive; they coexisted and complemented each other.

#### Maladaptive coping mechanisms.

Individuals experiencing infertility resorted to isolation and avoidance to cope with infertility‑related stressors such as societal stigma and emotional distress. Across multiple settings (Ghana, Ethiopia, and Nigeria), individuals withdrew from social gatherings out of fear of judgment and painful inquiries about their fertility status [6,8,63,65]. Avoidance often manifests as physical isolation, with individuals preferring to stay home or lock themselves away [41]. Quantitative findings concurred that people with infertility issues had high scores of active-avoidant syndromes, which were associated with lower levels of quality of life [63], and most of those engaging in escape-avoidance also exhibited mild depression [73].

Denial and defensiveness frequently emerged as maladaptive coping strategies among individuals facing infertility [82]. Both males and females often lied about their fertility circumstances to avoid scrutiny and retain a sense of control over their personal narratives [62] for instance, by arguing that they were still studying [81]. Others turned to substances, including alcohol, to escape their pain and frustration [72]. Such defensiveness functions as a protective response to infertility‑related stigma, allowing individuals to shield themselves from judgment and preserve social standing.

The review findings also capture some extreme suspicions, such as fear that childless individuals may steal other people's children [38,68]. Oti-Boadi and Asante (2017) found that negative religious coping was significantly correlated with age, duration of infertility, and elevated levels of somatization, depression, and anxiety [75]. The interplay of avoidance, denial, substance use, and negative religious coping underscores the need for supportive interventions that integrate care for psychological distress linked to infertility and the social stigma that fuels maladaptive responses.

## Discussion

There is an increasing number of studies in the last decade inquiring into the effects of infertility on mental health and relationship quality in SSA [27,28], yet this review identified several critical evidence gaps. This trend may reflect growing recognition of the burden of infertility; however, there remains a paucity of studies documenting interventions to mitigate its impact [26]. Studies in the review predominantly targeted women (66%; 38/58), perhaps highlighting the traditional focus on women around reproduction and infertility [83]. One-quarter of articles focused on both sexes (24%), and only 10% targeted men, implying that research on effects of infertility on mental health and relationship quality may be under-representing male perspectives and experiences, yet these are critical in designing interventions that fit the targeted populations.

Studies in this review affirmed the association of infertility with mental health experiences and relationship outcomes [23,38,42,43,62]. More particularly, the review findings were consistent on the emotional distress infertility exerts on individuals, further exacerbating existing tensions within relationships, leading to communication breakdowns and, in severe cases, separation or divorce [8,13,14]. Notably, there were few reports of the psychosocial support and therapy individuals and couples receive when seeking fertility-related services [12,21]. As such, these findings point to an essential programmatic gap in infertility management that integrates provision of proactive counselling and a range of psychosocial support services focused on improving communication and emotional resilience [84,85].

The review findings also suggested how societal stigma surrounding infertility is pervasive and manifests in different ways, including ostracization and discrimination from community activities, degrading insults, and abuse [37,57]. These expressions further triggered feelings of shame, blame, self-worthlessness, and actions including avoidance, isolation, social withdrawal, secrecy, and lying about their fertility situation [69]. Stigma drives women and men towards several forms of maladaptive coping mechanisms, sometimes harmful to their health and well-being. Support networks, such as peer-led groups that bring together individuals living with infertility (physically or virtually, like in WhatsApp groups), have been shown to offer safe spaces for sharing experiences and fostering connections [86]. Participants struggling with infertility shared their experiences in peer groups and emphasized the value and comfort derived from deep understanding and empathy when they shared experiences within the peer groups [40]. The empathetic connection helped individuals feel seen, understood, and less isolated in their struggles. Prior studies show that shared traumatic experiences foster deeper empathy and understanding, and that support from peers navigating similar journeys was particularly impactful.

However, more research and attention are required on interventions at the societal or community levels to mitigate infertility-related stigma, to enhance the lived experiences of individuals suffering from infertility, and to improve their physical and mental health [87].

Review findings also showed the intersection between infertility and experiences of violence, abuse, and diminished human rights of individuals [88]. Studies reported a heightened risk of experiencing various forms of emotional and physical violence due to infertility. Studies have suggested that marital counselling is among the effective interventions for couples with a history of IPV or psychological trauma and living with infertility [86]. A systematic review of structural interventions for intimate partner violence suggested integrating IPV screening and counselling within infertility clinics could help enhance early detection of IPV, and mitigation, including timely referrals for specialized care, such as psychological support groups or law enforcement, when necessary [89].

The findings showed that individuals with infertility relied on multiple therapy approaches, sometimes concurrently or sequentially, beginning with traditional and religious approaches, followed by professional medical care [90]. These healthcare-seeking patterns around infertility may partly reflect the conceptualization of infertility in many African societies as a non-medical issue that can be redressed using non-medical interventions [12,41,57]. Nonetheless, it is also primarily associated with the inaccessibility of fertility care, both due to cost and the availability of these services in much of SSA [19,21]. There is substantial literature on the unavailability of fertility care and how it diminishes human rights and reproductive justice - the right and ability to access infertility treatment and services, especially for the economically marginalized in societies [91].

The review findings suggest that health providers need to integrate a combination of resources when attending to patients seeking infertility treatments, including offering support towards spiritual, traditional, and biomedical care pathways that resonate with the personal beliefs and values of those faced with infertility. This holistic approach can enhance coping mechanisms, allowing individuals to feel more in control of their experiences and less stigmatized by societal pressures. Additionally, support systems, including healthcare providers and peer networks, are recommended as they play a crucial role in enhancing resilience and promoting mental well-being among people suffering from infertility.

Infertility can have wide-ranging implications for individuals and couples because it often affects emotional wellbeing, intimate relationships, social identity, and everyday functioning at the same time [12,14]. The effects may differ across contexts, but several common implications are consistently reported. The effects highlighted in this scoping review could also lead to broader outcomes: increased need for mental health and psychosocial support services or reduced healthcare-seeking due to fear, stigma or discrimination, and greater financial burden from expensive and prolonged treatment. It also affects family wellbeing, social participation, and life planning. Infertility should therefore be understood not only as a biomedical condition, but also as a social, psychological, and relationship issue with important implications for wellbeing and quality of life.

There is a critical gap in the development and testing of interventions that address the common relationship and mental health challenges associated with infertility in SSA [20]. Investment in such programs remains inadequate and contributes to poor outcomes related to infertility. In contrast, studies from high-income settings have reported several successful interventions that could be transferable and integrated into care systems within low- and middle-income settings with low resource inputs [12]. For instance, the Fertility Life Counselling Aid has shown great potential in addressing the psychological distress and stigma associated with infertility. The program follows a three-step process: *(1)* recognizing unhealthy thought patterns, *(2)* replacing them with healthier ones, and *(3)* practicing these positive thoughts and behaviors [92]. A context-specific counselling aid (adopted from FELICIA) could empower individuals dealing with infertility to develop healthier coping strategies and reduce stigma by fostering greater societal acceptance and understanding.

Talaei and colleagues confirmed that availing cognitive behavioral therapy to women living with infertility was associated with decreased depressive symptoms and psychological problems [93]. Other studies have confirmed the effectiveness of cognitive behavioral therapy on men [94]. Such interventions could optimize management of stress that is linked to infertility and potentially reduce the risk of relationship dissolution.

### Study limitations

This scoping review has several limitations. First, the review was limited to studies published from the year 2000 onward. This may exclude valuable insights from earlier research that could provide a broader context for understanding the evolving dynamics of infertility and its impact on relationships and mental health in the region. However, we also believe this timeframe covers enough research to provide a detailed scope of the state of evidence on this topic. This decision was mainly influenced by the need to be focused, practical, and pragmatic, given the scope the researchers could manage with available resources and time.

Additionally, the review predominantly focused on studies written in English, which may inadvertently overlook significant findings from research published in other languages, particularly in a multilingual region like SSA. We nevertheless made every effort to obtain studies from the francophone and other parts of Africa that were published in English.

Because scoping reviews do not typically include critical appraisal of the methodological quality of included studies, we could not systematically assess the strength and quality of the available evidence. A future systematic review incorporating formal quality and risk-of-bias assessments would help further evaluate the robustness of the evidence identified in this review.

Lastly, the review emphasizes published articles and excludes grey literature such as dissertations and reports from non-governmental organizations. This omission may result in a narrower perspective on the impact of infertility on mental health and relationship quality, as grey literature often contains valuable data that can illuminate local experiences and interventions not captured in peer-reviewed journals.

### Perspective on future research (research gaps)

This scoping review highlights several critical research gaps, with very few studies employing ethnographic approaches. Ethnographic research could offer deeper, longitudinal insights into individuals’ lived experiences and warrants further exploration. Notably, there is a scarcity of interventional studies aimed at addressing infertility and its effects on relationship quality and mental health. This lack of intervention-focused research limits the ability to identify effective support and care strategies. Additionally, geographic distribution of the existing literature suggests that many regions within SSA are underrepresented in research on the topic. These regions offer unique policies, health systems, and cultural contexts that shape experiences of infertility, and it is valuable to generate research and evidence from these settings. The review also notes the dearth of community-based quantitative studies, which are essential for understanding the broader societal implications of infertility. Addressing these gaps will be crucial for building the evidence base on infertility and its effects, potentially strengthening efforts to prioritize the topic in public policy discourses and increase investment towards greater access and affordability of fertility treatments in Sub-Saharan Africa.

## Conclusion

The scoping review revealed that infertility was closely associated with mental health and relationship problems, especially because infertility is often overlooked in health policies, programs, and services in sub-Saharan Africa. Many studies reported that people experiencing infertility face stigma, discrimination, anxiety, and depression. Infertility also affects relationships, with studies describing experiences such as infidelity, emotional and physical violence, separation, and divorce. The stigma around infertility can lead to social isolation and unhealthy coping behaviors. However, most studies focused mainly on women, with limited attention given to men’s experiences. In addition, some countries and regions in SSA remain underrepresented in the existing evidence on infertility. The scoping review highlights several critical research gaps and the need for broader social interventions that improve access to fertility services, strengthen social support, and promote healthy coping strategies. Counselling services and community-based programs are also needed to reduce stigma and protect the mental well-being of individuals affected by infertility.

## Supporting information

S1 Table Key search concepts and search strategy. (DOCX)

S2 Table Summary of review findings. (DOCX)

S1 Checklist PRISMA checklist - Preferred reporting items for systematic reviews and meta-analyses extension for scoping reviews (PRISMA-ScR) checklist. (DOCX)
