# Supplementary material for: Effects of infertility on relationship quality and mental health outcomes in sub-Saharan Africa: A scoping review
Source: PLOS Glob Public Health. 2026 Nov 10;6(11):e0005923. doi: 10.1371/journal.pgph.0005923 (PMC13750579; doi:10.1371/journal.pgph.0005923)
Supplement: Supplementary file 1 [file pgph.0005923.s001.docx]

**S1: Key Search concepts and their synonyms**

|  | **AND** | **AND** | **AND** | **AND** |
| --- | --- | --- | --- | --- |
|  | **Infertility** | **Sub-Saharan Africa** | **Relationship quality** | **Mental Health** |
| **OR** | Childlessness | East Africa | Intimate partner violence | Anxiety |
| **OR** | Sterile | Southern Africa | IPV | Depression |
| **OR** | Barrenness | West Africa | Domestic violence | Stigma |
| **OR** | Impotence | Central Africa | Marital dissolution |  |
| **OR** | Infecundity | (Angola OR Benin OR Botswana OR "Burkina Faso" OR Burundi OR "Cape Verde" OR Cameron "Central African Republic" OR Chad OR Comoros OR Congo OR "Democratic Republic of the Congo" OR "Cote d'Ivoire" OR "ivory coast" OR Djibouti OR "Equatorial Guinea" OR Eritrea OR Seatini OR Swaziland OR Ethiopia OR Gabon OR Gambia OR Ghana OR Guinea OR Guinea-Bissau OR Kenya OR Lesotho OR Liberia OR Madagascar OR Malawi OR Mali OR Mauritania OR Mauritius OR Mozambique OR Namibia OR Niger OR Nigeria OR Rwanda OR "Sao Tome and Principe" OR Senegal OR Seychelles OR "Sierra Leone" OR Somalia OR "South Africa" OR "South Sudan" OR Sudan OR Tanzania OR Togo OR Uganda OR Zambia OR Zimbabwe) | Divorce |  |
| **OR** | Primary infertility |  | Separation |  |
| **OR** | Secondary infertility |  |  |  |
| **OR** | Assisted Reproduction |  |  |  |
| **OR** | In Vitro Fertilization |  |  |  |
| **OR** | Fertility Clinics |  |  |  |

**Search term combination and databases**

|  | **Data base** | **Searched within:** | **Search term combination** | **Number of articles retrieved** | **General comments on database** |
| --- | --- | --- | --- | --- | --- |
| 1 | Pubmed | Title/Abstract MeSH Terms - sub-Saharan Africa, Africa | ((((((((((Infertility) OR (childless*)) OR (sterile)) OR (impoten*)) OR (infecundity)) OR ("primary infertility")) OR (infertile)) OR ("secondary infertility")) OR ((("Assisted Reproduction") OR ("In Vitro Fertilization")) OR ("Fertility Clinics"))) AND ((((((((((("Intimate partner violence") OR (IPV)) OR ("marital dissolution")) OR (divorce)) OR (separation)) OR ("domestic violence")) OR ("domestic abuse")) OR (anxiety)) OR (depression)) OR (stigma)) OR (stress))) AND ("sub-Saharan Africa" OR "East Africa" OR "Southern Africa" OR "West Africa" OR "Central Africa" OR Angola OR Benin OR Botswana OR "Burkina Faso" OR Burundi OR "Cape Verde" OR Cameron "Central African Republic" OR Chad OR Comoros OR Congo OR "Democratic Republic of the Congo" OR "Cote d'Ivoire" OR "ivory coast" OR Djibouti OR "Equatorial Guinea" OR Eritrea OR Seatini OR Swaziland OR Ethiopia OR Gabon OR Gambia OR Ghana OR Guinea OR Guinea-Bissau OR Kenya OR Lesotho OR Liberia OR Madagascar OR Malawi OR Mali OR Mauritania OR Mauritius OR Mozambique OR Namibia OR Niger OR Nigeria OR Rwanda OR "Sao Tome and Principe" OR Senegal OR Seychelles OR "Sierra Leone" OR Somalia OR "South Africa" OR "South Sudan" OR Sudan OR Tanzania OR Togo OR Uganda OR Zambia OR Zimbabwe) | 751-with English filter only | The broad results were giving close to 6500 articles which are not manageable. For that reason we have decided to introduce the relationship quality and mental health aspects to refine the search to manageable number. No time filters applied |
| 2 | Cochrane library |  | (((((((Infertility) OR (childless*)) OR (sterile)) OR (impoten*)) OR (infecundity)) OR ("primary infertility")) OR (infertile)) OR ("secondary infertility") in Title Abstract Keyword OR (("Assisted Reproduction") OR ("In Vitro Fertilization")) OR ("Fertility Clinics") in Title Abstract Keyword AND (((((((((("Intimate partner violence") OR (IPV)) OR ("marital dissolution")) OR (divorce)) OR (separation)) OR ("domestic violence")) OR ("domestic abuse")) OR (anxiety)) OR (depression)) OR (stigma)) OR (stress) in Title Abstract Keyword AND "sub-Saharan Africa" OR "East Africa" OR "Southern Africa" OR "West Africa" OR "Central Africa" OR Angola OR Benin OR Botswana OR "Burkina Faso" OR Burundi OR "Cape Verde" OR Cameron "Central African Republic" OR Chad OR Comoros OR Congo OR "Democratic Republic of the Congo" OR "Cote d'Ivoire" OR "ivory coast" OR Djibouti OR "Equatorial Guinea" OR Eritrea OR Seatini OR Swaziland OR Ethiopia OR Gabon OR Gambia OR Ghana OR Guinea OR Guinea-Bissau OR Kenya OR Lesotho OR Liberia OR Madagascar OR Malawi OR Mali OR Mauritania OR Mauritius OR Mozambique OR Namibia OR Niger OR Nigeria OR Rwanda OR "Sao Tome and Principe" OR Senegal OR Seychelles OR "Sierra Leone" OR Somalia OR "South Africa" OR "South Sudan" OR Sudan OR Tanzania OR Togo OR Uganda OR Zambia OR Zimbabwe | Cochrane reviews-430 | Same search approach as PubMed |
| 3 | JSTOR |  | First combination  Infertility OR childless* OR “Assisted Reproduction” OR “In Vitro Fertilization” AND “Intimate Partner Violence” OR depression AND “Sub-Saharan Africa” Second Combination  Infertility OR Baren* OR “Assisted Reproduction” OR “Fertility Clinics” AND “Intimate Partner Violence” OR anxiety AND “Sub-Saharan Africa” Third Combination  Infertility OR impoten* OR “Assisted Reproduction” OR “In Vitro Fertilization” OR “Fertility Clinics” AND “Intimate Partner Violence” OR Stigma AND “Sub-Saharan Africa” | Total =50   First combination = 38 results  Second Combination = 6 Results   Third Combination = 6 results | *Due to the database limitation, (having limited query at a time) we only focused on the key terms for the three combinations |
| 4 | ScienceDirect | Title, abstract, author-specified keywords | First combination  (Infertility OR childlessness OR "Assisted Reproduction" OR "In Vitro Fertilization" ) AND (“Intimate partner violence” OR depression ) AND ("sub-Saharan Africa")  Second combination (Infertility Or Barenness OR “Assisted Reproduction” OR “Fertility Clinics”) AND (“Intimate Partner Violence” OR anxiety) AND (“Sub-Saharan Africa”)  Third combination  (Infertility Or impotence OR “Assisted Reproduction” OR “InVitro Fertilization” OR “Fertility Clinics”) AND (“Intimate Partner Violence” OR Stigma) AND (“Sub-Saharan Africa”) | Total= 776 First combination = 646 results (English Filter)  Second combination = 52 results  Third combination= 78 results | *Due to the database limitation, (having limited query at a time) we only focused on the key terms for the three combinations |
| 5 | HINARI | Abstract | (((((((((Infertility) OR (childlessness)) OR (sterile)) OR (barrenness)) OR (impotence)) OR (infecundity)) OR ("primary infertility")) OR ("secondary infertility")) OR ((("Assisted Reproduction") OR ("In Vitro Fertilization")) OR ("Fertility Clinics"))) AND ("sub-Saharan Africa" OR "East Africa" OR "Southern Africa" OR "West Africa" OR "Central Africa" | 476 results - with English filter | The individual countries not listed |
