# Supplementary material for: Effects of infertility on relationship quality and mental health outcomes in sub-Saharan Africa: A scoping review
Source: PLOS Glob Public Health. 2026 Nov 10;6(11):e0005923. doi: 10.1371/journal.pgph.0005923 (PMC13750579; doi:10.1371/journal.pgph.0005923)
Supplement: Supplementary file 2 [file pgph.0005923.s002.docx]

**S2: Summary of review findings**

| **Author, Year** | **Country** | **Study objectives** | **Study design and data type** | **Setting and participants** | **Theme** | **Summary of findings** |
| --- | --- | --- | --- | --- | --- | --- |
| Elwell, 2022 | Malawi | To explore women's attitudes about infertility from the viewpoints of both women and healthcare professionals. | Cross-sectional; Qualitative | Healthcare: Female and Male | Mental health and separation/divorce | - Stigma & social exclusion: infertile women are mocked, gossiped about, labelled (e.g., witches or barren), and socially excluded - Emotional distress: Infertility brings intense emotional pain, stress, and pressure from peers and in-laws. - Gendered blame: Infertility blamed on women, equating womanhood with motherhood. - Marital strain: Women face verbal abuse and threats of divorce or polygamy |
| Groone et al.,2021 | Tanzania & Ghana | To assess infertility-related stress among women seeking fertility care treatment in a healthcare setting in central Tanzania and Ghana | Cross-sectional study; Quantitative | Healthcare; Female | Mental health | - Tanzania: highest infertility-related stress in the need for parenthood domain (FPI=45.09, SD=12.82 (being a parent as a primary/essential life goal) - Social concern (FPI=34.81, SD=13.51) (sensitivity to comments about infertility) - Relationship concern (FPI=31.33, SD=9.53) (feelings of isolation from family/peers) - USA: Infertility-related stress (mean FPI = 127.67, SD = 33.26) - Canada Infertility-related stress (mean FPI = 134.4, SD = 33.8) - Ghana Infertility-related stress (mean FPI = 172.5, SD = 34.2) |
| Ofosu-Budu et al., 2021 | Ghana | To explore ways of reducing stigma from infertility in Ghana | Phenomenology; Qualitative | Community; Female | Mental health | - Infertility linked to curses, witchcraft, abortion, reinforcing longstanding stigma - Beliefs contribute to social isolation and internalized hopelessness among affected |
| Akintayo et al., 2022 | Nigeria | Explore the effect of infertility on self-esteem and depression among infertile women in Nigeria. | Case-Control study; Quantitative | Healthcare: Female | Mental health and separation/divorce | - Depression- 17% among infertile women and 8% among fertile women - Low self-esteem- 19.4 score among infertile women vs 20.7 score among fertile women - Remarriage- (major depressive disorder, No =13, Yes=4), (No major depressive disorder, No=81, Yes =2) |
| Alhassan etal.,2014 | Ghana | Assess the prevalence and severity of depression in infertile Ghanaian women | Cross-sectional study; Quantitative | Healthcare: Female | Mental health | - Depression - 62.0% of infertile women had depression; 38.0% had no depression - Severity of depression: 40.0% had mild, whereas 22.0% had moderate depression |
| Oti-Boadi & Asante,2017 | Ghana | To describe infertile women's psychosocial health problems and their infertility-related beliefs | Descriptive correlational cross-sectional survey study; Quantitative | Healthcare (Assisted Reproduction Clinics in Ghana); Female | Mental health | - Infertile women had high levels of stress, low anxiety, social isolation, low perceived stigma, and high levels of depressive symptoms. - Beliefs that infertility has negative consequences were significantly related to infertility-related stress, social isolation, and depressive symptoms. - Belief that infertility could be managed by personal control was significantly related to lower levels of anxiety and perceived stigma. - Beliefs about consequences, illness coherence, and personal control explained significant proportions of variances in anxiety, stress, isolation, stigma, and depressive symptoms. |
| Annan‑Frey et al., 2023 | Ghana | To explore the lived experiences and coping strategies of infertile couples in Kumasi, Ghana | Phenomenology; Qualitative | Healthcare (3 Hospitals; Female/Male | Mental health | - Internalized stigma: "Life is not fair; I sometimes feel God shouldn’t have created me" - Enacted stigma: "I got married before a neighbour, but she gave birth almost immediately. Everyone is talking about me because I have kept long [in giving birth]." |
| Naab et al., 2019 | Ghana | Test the feasibility of a culturally adapted depression intervention among infertile women | Randomized Controlled Trial; Quantitative | Healthcare; Female | Adaptive coping mechanisms | - Individuals exposed to a psychosocial intervention (Oh happy day class) had better psychosocial health (reduced symptoms of depression at 6–12 weeks by an average of almost 11 points, and maintained the effect up to 24 weeks) |
| Hess 2018 | Mali | To examine infertility induced psychological distress and coping strategies among women in Mali. | Cross-sectional study, phenomenology; Mixed-Method Study | Healthcare; Female | Mental health and separation/divorce | - Emotional distress: Infertility caused deep grief, stress, anxiety, and fear of abandonment, often leading to internal conflict despite reassurance from partners. - Stigma & exclusion: Women face accusatory remarks, exclusion, and community judgement, reinforcing isolation and marginalisation. - Family pressure & marital breakdown: Intense family pressure contributes to relationship instability, including divorce or polygamy, creating further emotional pain. |
| Diallo et al., 2024 | Ghana | To explore the psycho-social burden of infertility in Cape Coast, Ghana | Phenomenology; Qualitative | Healthcare: Female and Male | Mental health | - Stress and frustration - Women experienced stress and frustration when community members offered unsolicited traditional “cures” for infertility - Intense grief/distress when confronted with others’ childbirth, often withdrawing to cry - Desperation- Women report engaging in unusual or extraordinary actions in their attempts to conceive, reflecting the intense pressure and desperation - Depression - Sadness, sleep difficulties, and disinterest in previously enjoyable activities. - Internalised stigma- Women internalized blame, with past experiences such as abortion intensifying guilt and avoidance of care, self-questioning and sadness - Enacted stigma- Women experience stigma in religious and family settings, where childlessness is framed as a curse or personal failure. - Emotional abuse- At work, infertile women were dismissed or devalued through assumptions that they lack caregiving responsibilities |
| Rufai et al. 2022 | Nigeria | Assess the link between family functionality and depression in Nigeria. | Cross-sectional study; Quantitative | Healthcare; Females with infertility | Mental health | - Depression- Most respondents (230, 55.4%) had no (minimal) depression, while 185 (44.6%) had depression; 135 (32.5%) respondents had mild depression, while 50 (12.1%) had moderate depression. None had severe depression |
| Omoaregba et al., 2011 | Nigeria | Determine the prevalence of psychological distress among women in Nigeria | Case control study; Quantitative | Healthcare; Female | Physical and emotional abuse | - Verbal and Physical abuse- Most (64%) reported experiencing some form of abuse (verbal and physical) because of their infertility. The commonest sources of abuse were from neighbours (15%), spouses’ relatives (14%), and their spouses (10%). |
| Naab et al., 2019 | Nigeria | To explore the psychosocial experiences of women with infertility in Zamfara | Exploratory; Qualitative | Healthcare; Female | Mental health, infidelity and separation and divorce | - Emotional distress: Infertility leads to anxiety, depression, anger, stress, forgetfulness, and difficulty concentrating, affecting women’s mental functioning and daily life. - Stigma and verbal abuse: Women face exclusion, ridicule, and degrading comments that reinforce feelings of inadequacy and social isolation. - Marital strain and infidelity: Infertility contributes to secrecy, lack of communication, extramarital relationships, and emotional disconnection within marriage. - Marital breakdown: Pressure from relatives results in divorce or polygamy, leaving women feeling rejected and unstable. |
| Dyer et al., 2009 | South Africa | To assess psychological distress in infertile men in South Africa | Case-control study; Quantitative | Healthcare; Male | Mental health | - Depression- prevalence of depression was 58.7% (study group) and 50.1% (control group) - Anxiety- prevalence of depression was 56.2% (study group) and 47.1% (control group) - Anger- prevalence was 55.7% in the study group and 50.6% in the control |
| Fledderjohann 2012 | Ghana | This paper explores the implications of infertility for women in Ghana, West Africa. | Cross-sectional study; Quantitative | Healthcare; Female | Mental health and Physical and emotional abuse | - Sadness and insomnia– “I’ve been crying always about things people say and what they do. When you go home, you can’t even sleep”. - Anxiety- "Everyone wants to give you advice, but in the end, you are alone in this infertility, and there is a lot of anxiety". - Enacted stigma- "They don’t treat you well.., they keep their children away from you, and if you want to send someone else’s child, the mother won’t allow it. They’ll say send your own child". - Verbal abuse- "I can’t go to my husband’s family because they insult me, they laugh at me... they will say things to make you hang yourself ". - Pressure from family and friends- "My friends understand, but my mother-in-law wants a grandchild. She accuses me, and this could destroy the marriage, even though the problem could be with the man". |
| Moyo, 2013 | Zimbabwe | The present study sought to remedy social and psychological impacts of male infertility for the affected individuals or couple in Zimbabwe that might influence policy formulation about the condition | Ethnography; Qualitative | Community; Male | Mental health, infidelity and Physical and emotional abuse | - Suicidal ideation- "Infertility provokes feelings of inadequacy and paucity, coupled with renunciation of the problem, leading to suicide and suicidal melancholy in some men". - Sadness- "Married men with children are traditionally prefixed as father of Tadiwa, or Simbiso, Nyasha, Chipo. But my situation is very painful. I am now 60 years old, and everyone just addresses me as Zivo. I wish I could be called ‘Father of Monica". - Enacted stigma- "I was very humiliated when I sent my neighbour’s child to the shops, and the mother told me to impregnate my wife and have my own children to send on errands. This was hurtful because this was said in front of a large crowd". - Infidelity- "men engage in extra-marital relationships, hoping to impregnate one woman along the way, and this exposes them to risks of STIs and HIV" - Emotional abuse- Infertile men experience deep emotional pain, humiliation, and public ridicule, and endure hurtful jokes about their masculinity, and try to protect their partners emotionally while silently carrying stress and shame |
| Ameh et al., 2007 | Nigeria | Assess the burden of domestic violence among infertile women at a Clinic in Nigeria | Cross-sectional study; Quantitative | Healthcare; Female | Mental health | - Psychological distress- 203 (79.9%) of the participants had psychological distress, while 51 (20.1%) had no psychological distress |
| Tabong & Adongo 2013 | Ghana | This study, therefore, explored the experiences of infertile couples in Northern Ghana using the Upper West Region as a case study. | Exploratory; Qualitative | Community: Female and Male | Infidelity and Reduced sexual satisfaction | - Sadness- “I weep any time my neighbour beats her children, and they are crying because this reminds me of my childlessness despite my unconditional love for children”. - Enacted stigma- “Even though I am married to the firstborn in this family, my contribution is valueless because I have no child. I only listen during family meetings because am afraid to be insulted by other relatives”. - Reduced sexual dissatisfaction- “I have not had sex with my wife for more than six months now because it is a waste of energy. . . why continue to have fruitless sex with your wife. Akpetseshie (local gin) is now my wife; take it and forget the frustration”. - Infidelity- I endured painful rituals after a secret extramarital affair to save my marriage, but I was discovered and punished |
| Abubakar et al., 2021 | Nigeria | To determine the impact of family social support on the psychological well-being of infertile women | Cross-sectional Study; Quantitative | Healthcare; Female | Separation/divorce | - 110 (43.3%) were from functional families vs 89 (35.0%) from moderately dysfunctional families and 55 (21.7%) from highly dysfunctional families. - There was a significant association between family function and psychological distress |
| Dyer et al., 2005 | South Africa | Assess psychological distress among women suffering from couple infertility in South Africa. | Case-control study; Quantitative | Healthcare; Female | Physical and emotional abuse | - Intimate partner violence- 17 (14.5%) reported intimate partner physical violence - Verbal and emotional abuse- 29 (24.8%) reported partners used verbal and/or emotional abuse and 26 women reported such abuse from their in-laws. |
| Donkor et al., 2017 | Ghana | The purpose of this study was to explore the psychological experiences of women with infertility | Exploratory; Qualitative | Healthcare; Female | Mental health and reduced sexual satisfaction | - Loneliness- “My sister-in-law is emotionally abusive. Whenever we disagree, she will remark, “can’t you see I have brought children to this home? Where are yours? - Anxiety- "I get very anxious when I see all who got married after me are having children. I am the only one left behind. I am aging… I am approaching menopause soon". - Depression- “I am always depressed. After three years of marriage, society expects that I get pregnant or carry my own baby and I have failed to achieve these". - Sexual dissatisfaction- “I don’t enjoy sex with my husband. After years of regular intercourse, I am not pregnant. Sex has no need as it doesn’t lead to childbirth". |
| Anokye et al., 2017 | Ghana | To determine the psychosocial effects of infertility among couples | Descriptive; Quantitative | Healthcare; Female and Male | Mental health and Physical and emotional abuse | - Depression- 8% indicated that infertility led to low self-esteem, while 17% mentioned distress and 15% depression - Intimate partner violence, abuse and divorce- 41% experienced verbal and physical abuse, and 3% indicated that it had led to marriage breakdown (divorce). |
| Dhont et al., 2010 | Rwanda | Explore the consequences of infertility for men and women in Rwanda | Case-control study/Phenomenology; Mixed-Method Study | Healthcare: Female and Male | Separation/divorce, infidelity, mental health, Physical and emotional abuse | - Stigma: I am openly mocked and labelled, which reinforces my social exclusion - Marital instability: Infertility increases likelihood of relationship breakdown - Violence: I face higher risks of physical abuse, threats, neglect, and loss of support - Infidelity & Relationship Strain: Infertility is linked to increased extramarital relationships, further weakening trust and stability. |
| Donkor & Sandall 2007 | Ghana | Assess the extent to which Ghanian women seeking infertility care perceived themselves as stigmatised | Cross-Sectional study; Quantitative | Healthcare; Female | Mental health | - Stigma – 253 (41%) experienced severe stigma - Stress- Mean value of infertility-related stress was 172.5 (S.D.=34.2) - Relationship between stress and stigma - Women with severe levels of perceived stigma had the highest mean value for infertility-related stress |
| Kok, 2009 | Malawi | To construct practices related to infertility in Malawi as cultural or normative | Ethnography; Qualitative | Community: Female and Male | Infidelity | - People experiencing infertility construct polygamy and extramarital affairs as culturally and normatively required, `automatic' and normal solutions for fertility problems and play down people's accountability for these practices. - These perspectives facilitate permission for extramarital affairs and polygamy |
| Yusuf et al., 2012 | Nigeria | To determine the socio-demographic correlates of psychological distress among infertile males | Cross-sectional study; Quantitative | Healthcare; Male | Mental health | - Depression and anxiety: 23 (28.4%) were psychologically distressed. The most common form of psychological distress was depression (14 (17%)); 9 (11%) had anxiety. |
| Iliyasu et al., 2016 | Nigeria | Determine the prevalence of and risk factors for intimate partner violence among women in Nigeria. | Cross-sectional study; Quantitative | Healthcare; Female | Physical and emotional abuse | - Intimate partner violence and verbal abuse- 134 (36%) experienced at least one form of IPV, and of these, 126 (94%) experienced psychological violence, 111 (83%) experienced sexual violence, 47 (35%) were verbally abused, and 25 (19%) experienced physical violence |
| Bornstein et al., 2020 | Malawi | To understand the production and impact of infertility-related stigma in rural Malawi | Cross-sectional study; Qualitative | Community: Female and Male (51% were female and 49% were male) | Mental health, physical and emotional abuse, Infidelity | - Enacted Stigma- People don’t respect a man without children; they just use first names. - Internalized stigma - “I constantly feel targeted, assuming others are laughing at me.” - Labelling- "*Alibe nsalu ya lekaleka* - A childless woman cannot fulfil her role as a mother.” - Verbal abuse- “He insults me and calls me names like ‘you dog’ or ‘you are very stupid.’ “He feels no need to keep me, and that I must leave.” - Infidelity- Infertility breeds doubt, leading both partners to seek other relationships. - Extramarital relationships - Her husband admitted impotence and suggested hiring a man to impregnate her, but she refused due to health and moral concerns, left the marriage, and later remarried and had three children. - Extramarital partners (“fisi”) were sometimes arranged as a last resort; chosen for proven fertility and kept secret to preserve the appearance of a normal marriage. |
| Kyei et al., 2021 | Ghana | To document beliefs about childbearing/children and the psychosocial implications of infertility in Ghana | Exploratory; Qualitative | Healthcare; Female and Male | Mental health and Separation/divorce | - Emotional distress: I experience constant worry, anxiety about time and age, frequent crying, and deep hopelessness due to childlessness. - Severe psychological impact: Stigma and repeated disappointments push me toward thoughts of ending my life. - Stigma & exclusion: I am excluded from social and family events and feel unvalued in my community because I have no child. - Marital instability: My relationships are strained or end in divorce because I am childless |
| Esan et al., 2022 | Nigeria | To explore the challenges infertile women and their partners experience in Nigeria | Not reported; Qualitative | Healthcare: Female and Male | Adaptive coping mechanisms and Infidelity and separation/divorce | - Separation- “I told you he left me, he left me and I’m still alone - Infidelity- He had another woman because I couldn’t give him a child. - Marriage not affected - I thank God—despite eight years without conceiving, it has not affected my marriage. |
| Folkvord et al.,2005 | Zimbabwe | To explore factors associated with male infertility and distress | Cross-sectional study; Quantitative | Healthcare; Male | Mental health and separation/divorce | - Depression- 30% reported signs of mild depression, such as insomnia - 70% felt uncomfortable about their infertility. One-third had relational problems, and one-third have problems with the in-laws |
| Dorcas Ofosu-Budu et al., 2021 | Ghana | To explore the explanations for infertility among involuntarily childless women in Ghana | Phenomenology; Qualitative | Community; Female | Adaptive coping mechanisms and maladaptive coping mechanisms | - Infertile women attributed their condition to supernatural factors - Such explanations were assigned to conditions they couldn’t understand - Other explanations of infertility were medical, such as hormonal imbalance. |
| Njogu, Anne, et al. 2023 | Kenya | Examine effects of self-compassion training (videos versus digital stories) on reducing anxiety and depression | Randomized Controlled Trial; Quantitative | Healthcare; Female | Adaptive coping mechanisms | - Participants experienced significant reductions in anxiety and depression - Participants experienced significant increase in self-compassion and infertility self-efficacy - The interventions did not change the pregnancy rates. |
| Aduloju et al.,2015 | Nigeria | Evaluate the prevalence and predictors of intimate partner violence among infertile women | Cross-sectional; Quantitative | Healthcare; Female | Mental health and physical and emotional abuse | - Enacted stigma- “I used to feel ashamed; people mocked me, excluded me, and treated me as if I didn’t belong because I had no child.” - Physical violence- infertile women face notable physical and sexual violence - Emotional violence – psychological violence was the most common form experienced |
| Dierickx et al., 2018 | Gambia | Explores the implications of infertility on women’s lives in urban Gambia | Cross-sectional; Qualitative | Community; Female | Mental health, separation/divorce, physical and emotional abuse | - Stress- “My husband neglects me, and I return to my family, they tell me to be patient, but it is painful and overwhelming.” - Divorce- A woman left her marriage after ten childless years due to stigma and in-law pressure, choosing independence over continued trauma. - Pressure from family- my husband’s family pressured him to divorce me or take a second wife, and living with them led to constant conflict. - Intimate partner violence - I endured years being childless while my husband beat me repeatedly, telling me I wasn’t a real woman |
| Dierickx et al., 2021 | Gambia | This study explored men’s aetiological knowledge, views and experiences of infertility in the Gambia | Exploratory; Qualitative | community; Male | Mental health | - Sadness- “I feel ashamed when I compare myself to my younger siblings with children, and it makes me feel like I don’t belong.” - Enacted stigma- 'They call you, ‘oo wonna gorko’ (you are not a man), and call you with your wife’s name.’ |
| Dierickx, 2020 | Senegal | This study provided a holistic understanding of the experiences of women with infertility living in a rural community in Casamance (Senegal) | Cross-sectional study; Qualitative | community; Female | Mental health and separation/divorce | - Sadness- I felt sad—I even lost weight from constantly thinking about not having a child, and I envied other women who had theirs. - Verbal abuse- People called me a witch and said I was barren, that I would never have a child - Pressure from family and friends- My husband’s family pushed him to take another wife because they believed I wouldn’t have many children, even after I had two. |
| Hollos &Whitehouse, 2014 | Nigeria | To study the life course consequences of infertility in a Nigeria | Ethnography; Qualitative | community; Female | Mental health | - Childless women were characterized as “useless ” - Even with wealth or education, infertility was frowned upon - Respect was given to women who had even one child, even if that child died. - The biological process of gestation confers adulthood on women, allowing them to undergo initiation and to function as mature individuals. |
| Teklemicheal et al., 2021 | Ethiopia | Determine the prevalence of psychological stress among infertile women in Ethiopia | Cross-sectional study; Quantitative | Healthcare; Female | Mental health | - Stress- Prevalence of infertility-related stress: 93%; 47% reported a moderate to severe degree of stress |
| Sulyman and Kuranga, 2022 | Nigeria | To determine the prevalence of depression and associated factors among women attending infertility clinics | Comparative analytical study; Quantitative | Healthcare; Female | Mental health | - Depression- Prevalence of depression 52% (104 in the case group) had clinical depression. Only 9.5% (19/200) in the control group had depression. |
| Rooij et al.,2020 | Ghana | Explore infertility stigmatization and the quality of life among women and men | Cross-sectional study; Quantitative | Healthcare; Female and Male | Mental health | - Enacted stigma- Because of my infertility, people treat me as inferior and look down on me, and this stigma worsens my quality of life while leading me to withdraw and avoid others. |
| Adane et al., 2024 | Ethiopia | The main objective of this study was to explore the psychological and coping strategies of infertile women in Bichena town, Ethiopia | Phenomenology; Qualitative | Community; Female | Mental health and separation/divorce | - Psychological distress and insomnia:” I feel overwhelmed by constant stress, worry, sadness, and anger, which affects my emotions and daily functioning. My sleep is disrupted, and I lack the concentration and ability to carry out even simple tasks.” - Stigma & social blame: I am blamed, misunderstood, and stigmatized by family - Marital breakdown: Constant stress and lack of support led me to leave the relationship to find peace and regain control of my life.” |
| Tilson & Larsen, 2000 | Ethiopia | To determine factors influencing the risk of divorce in Ethiopia | Cross-sectional study; Quantitative | Community; Female | Separation/divorce | - Divorce- 95% of women who are childless divorced within 20 years, 85% divorced within the first 5 years. |
| Olamijuwon et al., 2020 | Cameroon, Gabon, Lesotho, Liberia, Sierra Leone | To investigate the relationship between involuntary childlessness and marital infidelity | Cross-sectional study; Quantitative | Community; Female | Infidelity | - Infidelity- Cameroon: prevalence of marital infidelity among childless (16%) vs only 5% among non-childless women - Sierra Leone: Marital infidelity among childless women was twice that of non-childless women - Gabon, Lesotho, and Liberia- no statistical evidence that the prevalence of marital infidelity differed between non-childless and involuntarily childless married women. |
| Fledderjohann, 2017 | Ghana | To examine infertility not only in marital unions, but also in non-marital sexual unions. | Retrospective cohort study;  Quantitative | Community; Female | Adaptive coping mechanisms | - Positive relationship between infertility and relationship instability for both married women and those in non-marital sexual unions |
| Oladeji & OlaOlorun,2017 | Nigeria | To determine the prevalence of depression among infertile women in Nigeria. | Cross-Sectional Study; Quantitative | Healthcare; Female | Mental health | Depression- prevalence of depression: 53%. |
| Howe et al., 2020 | Zambia | This article seeks to fill this gap by qualitatively describing the ways in which infertility in Zambia is socially and culturally loaded for both men and women | Ethnography; Qualitative | Healthcare; Female and Male | Mental health, Physical and emotional abuse, Separation/divorce | - Sadness- I feel deeply hurt when I’m called barren, and seeing others with children makes me question myself; their mockery worsens my pain. - Enacted Stigma- In my culture, respect comes with having a child—without one, I’m excluded, overlooked, and made to feel less valued. - Intimate partner violence- Women described abuse and coercion as normalized—being forced into sex, beaten, or driven from their homes due to infertility, with such violence often seen as expected or even a sign of love. - Chased away- In my second marriage, after seven years without a child, my husband said I wasn’t a real woman and chased me away. |
| Adelosoye et al.,2020 | Nigeria | To assess the impact of family function and husband support on depression severity among women with infertility in Nigeria | Cross-Sectional Study-descriptive; Quantitative | Healthcare; Female | Mental health | Infertility, depression and a functional family   - Prevalence of depression - 43% (145/337). 64% (93) had mild depression, 30% Moderate depression, and 5% severe depression - Half (48.9%) of depressed women have dysfunctional families, versus 38% women with functional families. - The majority of non-depressed (62.0%) had functional families |
| Ojo et al., 2017 | Nigeria | To evaluate depression in a group of infertile women | Not reported; Quantitative | Healthcare ; Female | Mental health | Depression: 15 (18%) of those with infertility were depressed versus 17 (24) with secondary infertility |
| Naab & Kwashie 2018 | Ghana | To explore the concerns of men with infertility about the blame for male infertility falling on their wives. | Exploratory; Qualitative | Healthcare; Male | Mental health | - Emotional Distress (Worry, Sadness, Anger): I feel constant worry, hurt, and frustration, which often leads to sadness and conflict in my home. - Severe Psychological Strain (Suicidal Thoughts): The pressure of infertility can overwhelm me to the point of considering ending my life. - Guilt & Compromise: I feel guilty and even consider extreme solutions, like allowing my wife to have a child with another man. - Loss of Control & Powerlessness: I feel excluded from decisions about my marriage and fertility, leaving me frustrated and powerless. |
| Awoyinka & Ohaeri, 2014 | Nigeria | Assessed depression and coping strategies among women with infertility in Ibadan. | Cross-Sectional Study-Descriptive; Quantitative | Healthcare ; Female | Mental health and separation/divorce | - Depression- Mild depression-48.1%, moderate -30.4%, severe depression-21.5% - Family pressure - 56 (71%) reported that grandparents express their desire for grandchildren. - Separation /Polygamy- One-third reported temporary separation or polygamy, while nearly half reported threats of divorce from their husbands and relations. |
| Nyarko and Hubert2015 | Ghana | This study examined the effects of infertility on marital relationships among fertility clients in Ghana. | Cross-sectional study; Quantitative | Healthcare; Female and Male | Mental health, reduced sexual satisfaction, separation/divorce | - Suicidal ideation - about one in ten reported contemplating suicide. - Psychological Trauma- 88% experienced psychological trauma due to infertility. - Guilt- Nearly half (45 %) reported blame of self for the infertility - Divorce- 40% reported that their partners threatened them with divorce - Sexual Life Affected- Infertility negatively affected the sexual life of participants, 64% reported that sexual intercourse was no longer fulfilling or enjoyable |
| Nieuwenhuis et al., 2009 | Nigeria | This study explored the impact of infertility on infertile men and women in Ibadan, Nigeria. | Cross-sectional study; Qualitative | Community; Female and Male | Mental health, infidelity, separation/divorce | - Worry- Being childless weighs heavily on me—I constantly worry, and sometimes feel that if I had children, my marriage would be happier - Enacted stigma- In Yorubaland, a home without children is incomplete, and a woman who cannot conceive is often viewed as a failure. - Remarriage and infidelity- Communities often urge the husband to take another wife, while some advice the woman to try another partner to determine who is “at fault.” |
| Makanjuola et al.,2010 | Nigeria | Factors associated with psychiatric morbidity in infertile women | Retrospective case control; Quantitative | Healthcare; Female | Mental health | - Depression and Anxiety- persons with infertility - depressive illness (38%), while 11% had generalized anxiety disorder |
| Dyer et al., 2002 | South Africa | Explores the concerns and experiences related to involuntary childlessness of infertile women in South Africa. | Cross-sectional study; Qualitative | Healthcare (infertility clinic in a tertiary referral centre); Female | Mental health, separation/divorce, physical and emotional abuse | - Severe emotional distress: I experience deep sadness, anger, hopelessness, and at times thoughts of ending my life due to the pain of childlessness. - Relationship strain: Infertility leads to conflict, abuse, and breakdown of trust - Stigma: I face ridicule, verbal abuse, and cultural stigma, leaving me feeling worthless - Constant pressure and fear of abandonment keep me anxious, sleepless, and uncertain |
| Upkong & Orji,2006 | Nigeria | Determine the prevalence of psychiatric morbidity in women suffering from infertility. | Cross-sectional study; Quantitative | Healthcare; Female | Mental health | - Depression and anxiety- Prevalence of psychiatric morbidity was 46% among infertile women (anxiety (38%) and depression (43%) - Childlessness and poor spousal support increase the risk of depression, while pressure to pay for treatment and thoughts of adoption add anxiety. |
| Sulyman et al.,2019 | Nigeria | Determine prevalence of anxiety and depressive disorders among women with infertility in Nigeria | Cross-sectional study; Quantitative | Healthcare ((Public Hospital); Female | Mental health | - Depression and Anxiety- 85 (41%) had anxiety and/or depressive disorders; 57 (28%) had anxiety disorders, while 53 (26%) had depression. - Enacted Stigma- 75 (36%) reported stigmatizing behaviours from husbands/relatives |
| Aduloju et al.,2018 | Nigeria | To examine the quality-of-life scores among fertile and infertile women | Cross-sectional study; Quantitative | Healthcare; Female | Mental health | - Psychosocial challenges- Childlessness lowers quality of life—affecting both physical, emotional, and mental health. |
| Balandya et al.,2024 | Tanzania | Estimate the prevalence of infertility and examine its association with intimate partner violence | Cross-sectional study; Quantitative | Other (Data used from DHS 2022); Female | Physical and emotional abuse | - More than one-third (38%) of women experienced at least one form of IPV |
| Maduka et al.,2025 | Nigeria | Investigate the prevalence and predictors of IPV among women seeking infertility in Nigeria | Cross-sectional study; Quantitative | Healthcare; Female | Physical and emotional abuse | - Prevalence of IPV among infertile women was 44.7%. The most common type of IPV was psychological abuse (36.7%), and verbal abuse (32%). |
| Arhin, 2025 | Ghana | To explore the coping strategies employed by couples after unsuccessful infertility treatment. | Exploratory; Qualitative | Healthcare; Female (12) and Male (6) | Mental health | - Poor concentration - I have struggled with infertility —it leaves me emotionally drained and unable to focus. My heart feels heavy and distracted. |
| Esan et al.,2025 | Nigeria | Investigate depression and support among infertile women in Nigeria, | Cross-sectional; Quantitative | Healthcare; Female | Mental health | - 90 (24.2%) had a mild depression, 36(9.7%) had a borderline clinical depression, 68(18.3%) had a moderate depression, 18(4.8%) had a severe depression, and 14(3.8%) had an extreme depression. |
| Mashaah et al., 2024 | Zimbabwe | To explore the  psychological, social, and cultural aspects of  female infertility | Phenomenology; Qualitative | Traditional Health Practitioners (THPs); Female | Mental health, Separation/divorce, physical and emotional abuse | - Stigma & social exclusion - I am judged, mocked, and excluded because I do not have children. I feel intense pressure to have a child to gain acceptance - Loss of identity & respect- Without children, I feel undervalued and seen as incomplete in my community. I withdraw from others to avoid shame, questions, and humiliation. - Marital strain- Infertility created tension, mistrust, and he resorted to secret arrangements to achieve pregnancy, leading to separation. - Family pressure - Relatives pressure and blame me because of childlessness. - Violence - I experience emotional and sexual abuse linked to my inability to conceive. |
| Adda et al., 2025 | Ghana | To understand the psychosocial challenges faced by women with infertility in Ghana | Cross-sectional study; Qualitative | Health Facility; Female | Reduced sexual satisfaction, separation/divorce | - Social pressure- I am constantly questioned and gossiped about because I did not conceive soon after marriage. I worry, panic, and have sleepless nights. - Emotional pain- Without children, I feel like I do not belong and fear losing my place - Stigma - I am told I am not a “real woman” without children. I am punished for infertility. - Sexual disengagement - I feel intimacy is pointless because it will not result in a child. - Marital conflict: My husband’s frustration over childlessness led to emotional strain. |
| Araya et al., 2024 | Ethiopia | To explore the lived experience of women living with infertility in Ethiopia. | Interpretative phenomenological; Qualitative | Health Facility; Female | Adaptive coping mechanisms, mental health | - Anxiety: I feel constant worry and stress, I don’t understand why I can’t conceive. - Fear of Loss: I fear losing my husband and feel like a failure, with each menstrual cycle. - Self-doubt: I question my womanhood when I compare myself to other women. - Hope & Meaning-Making: Despite the pain, I turn to faith and keep hoping for a child. |
| Musa et al., 2025 | Nigeria | Determine the challenges & coping strategies used by clients with infertility | Cross-Sectional study; Quantitative | Health Facility; Female | Separation/divorce | - The majority (61.7%) reported a change of behaviour from their partners, and more than half (54.2%) reported marital discord. |
| Beyene et al., 2025 | Ethiopia | To determine the prevalence of depression among infertile women | Cross-sectional study; Mixed methods | Medical College; Female | Mental health | - 147 (42.1%) had depression; nearly half (81/147, 55.1%) had mild depression, and one‐third (43/147, 29.2%) had moderate depression. |
| Behude et al., 2025 | Ethiopia | Explore the psychosocial experiences and coping strategies of infertile  couples in Addis Ababa, Ethiopia. | Descriptive phenomenological research design; Qualitative | Health Facility; Both male and female | Mental health and Separation/divorce | - Emotional distress: Multiple treatment failures lead to sadness and depression - Anxiety: Fear of childlessness and social expectations create intense, constant anxiety. - Stigma & social judgment: Childlessness leads to feeling incomplete or defective - Marital strain: Infertility creates distance, blame, and conflict, weakening communication and intimacy in relationships. |
| Pratt et al., 2025 | Uganda | Explored stigma experiences and factors impacting engagement in HIV and reproductive care | Cross-sectional study; mixed-methods Qual-Quant study | Health Facility; Males and females | Mental health and Separation/divorce | - Gendered blame: Women are blamed, while men’s infertility is rarely acknowledged - Loss of social status: Childlessness seen as incomplete, “half,” or not a real man or woman. - Marital instability: Infertility results in divorce, remarriage, or women driven from home. - Desperation: Individuals seek other partners to confirm fertility and restore acceptance. |
| Awodele et al. 2025 | Nigeria | Explored the psychosocial and emotional effects of male infertility in Nigeria | Not reported; Qualitative | Health Facility; Male | Mental health and Separation/divorce | - Emotional distress: Infertility causes intense worry, overthinking, and emotional strain, sometimes leading to severe outcomes like depression or suicidal thoughts. - Social isolation: I feel unable to engage socially or move freely with others - Family instability: Infertility created tension, disagreements, and broader family problems - Blame & masculinity pressure: Male infertility brings shame, fear of abandonment |
