# Supplementary material for: Effects of infertility on relationship quality and mental health outcomes in sub-Saharan Africa: A scoping review
Source: PLOS Glob Public Health. 2026 Nov 10;6(11):e0005923. doi: 10.1371/journal.pgph.0005923 (PMC13750579; doi:10.1371/journal.pgph.0005923)
Supplement: Supplementary file 4 [file pgph.0005923.s004.docx]

The Chief Editor

PLOS Global Public Health

January 15, 2026

**RE:**

**Response to preliminary screening of our manuscript entitled “Effects of infertility on relationship quality and mental health outcomes in sub-Saharan Africa: a scoping review” (**PGPH-D-26-00060)

We thank the PLOS Global Public Health journal editorial team for the prompt review and suggested changes. We have addressed the two points raised, as documented below.

1. Your manuscript is a Scoping Review. Please change the article type to 'Research Article', and ensure all submission questions are completed, noting the change in publication fees associated with Research Articles.

This has been done

2. We note that this manuscript is a scoping review; our author guidelines require that you upload a PRISMA-ScR checklist as supporting information. Information about the PRISMA guidance and blank checklists can be found here: <https://www.prisma-statement.org/scoping>).

We have included the PRISMA-ScR checklist as supporting information

Kind regards,

Kenneth Juma

On behalf of all (co) authors
