# Supplementary material for: Effects of infertility on relationship quality and mental health outcomes in sub-Saharan Africa: A scoping review
Source: PLOS Glob Public Health. 2026 Nov 10;6(11):e0005923. doi: 10.1371/journal.pgph.0005923 (PMC13750579; doi:10.1371/journal.pgph.0005923)
Supplement: Supplementary file 6 [file pgph.0005923.s006.docx]

**PLOS Global Public Health: PGPH-D-26-00060**

**Effects of infertility on relationship quality and mental health outcomes in sub-Saharan Africa: a scoping review**

Thank you for submitting your manuscript to PLOS Global Public Health. After careful consideration, we feel that it has merit but does not fully meet PLOS Global Public Health’s publication criteria as it currently stands. Therefore, we invite you to submit a revised version of the manuscript that addresses the points raised during the review process.

We thank the editor and reviewers for the timely evaluation and the opportunity to resubmit this manuscript. We have addressed all editorial and reviewer comments in detail within the letter of response, with revisions clearly indicated by page and line numbers and highlighted throughout the revised manuscript. We have submitted both the highlighted and the clean revised manuscript.

**Reviewers' comments:

Reviewer #1:**

I appreciate the authors for undertaking this work, and the editor for the opportunity to review this manuscript on a very important topic of the effects of infertility in sub-Saharan Africa. Below are my observations and comments.

We thank the reviewer for the thorough assessment of the manuscript and for the feedback. We have carefully addressed all comments and questions raised to the best of our ability and hope this feedback has greatly improved this revised version of the manuscript.

| **Reviewer comment** | **Response** |
| --- | --- |
| **Abstract** | |
| 1. Include the target population in the abstract to help the reader start off with the right perspective. | We thank the reviewer for pointing this out. We have revised the abstract and added details on the target population.  See the additions on page 7, line 123-124; and page 10, line 191-195. |
| ***Background***: | |
| 2. The aim of this scoping review was to examine the effects of infertility on relationship quality and mental health outcomes in SSA. It would help to specify the target population for the primary studies reviewed. | This has been addressed by clarifying that the target population was all individuals (male or female) living with infertility in sub-Saharan Africa.  The details are in page 10, line 191-195; and also in page 7, line 123-124. |
| 3. The search strategy is satisfactorily elaborated. | We are incredibly thankful to the reviewer for this generous comment, and we appreciate this feedback. |
| ***Methods*** |  |
| 4. Assessment of methodological quality reported as not done. Please add a citation supporting this position, if any. | We appreciate the reviewer's comment. We have provided the necessary citations to support our approach, that a scoping review does not necessarily require assessment of methodological quality. See ref #35; on page 14, line 264-266. |
| ***Results*** | |
| 5. Figure 1 (PRISMA flow diagram). 5. Be explicit on the reasons for exclusion of papers, either within the diagram or the narrative that follows, with specific number of papers excluded at each stage. | This has been addressed and all the exclusion reasons at each stage added. See page 15, line 269-274.  We have also updated the PRISMA flow diagram. |
| 6. Table 3 showing the characteristics of the study included can be enhanced by, combining some aspects in same row (e.g. Author, country, aim of the study) and adding important information such as study design, measures used and key findings. This would add transparency and ensure credibility of the results reported. A table in landscape would accommodate key information on the same page. Key information for each study in the same row would enhance accessibility for the reader. For example, a reader would see which study had only females, males or both at a glance. | We appreciate this comment and this being a scoping review; we feel Table 3 summarising study characteristics is valuable in providing an overarching summary of what studies exist, summary of characteristics and study designs used.  Nevertheless, in appreciating the reviewers comments, we have also added an Appendix table S3 on summary of review findings where we clearly give a brief summary of results - study by study - included in this review. See Appendix S3 |
| 7. Key themes: As noted above, it would help to first summarize the key findings per paper in table 3 before showing the themes that emerge from the review. In its current form we don’t know for example how many papers reviewed reported on aspects of infertility and relationship quality and it therefore remains unclear how this theme emerged from your review. | This has been addressed in Appendix S3 that provides a summarised study by study findings. |
| 8. A descriptive account of the results from each paper/country rather than a clear synthesis of similar results is given. Since the review includes papers employing diverse study designs, it would help to see how the authors try to integrate findings from different designs. | We really appreciate the reviewer for raising this point. We have addressed this both in the methods and results sections of the manuscript.  We first clearly indicated that we conducted an iterative narrative synthesis and integrated the quantitative and qualitative findings (abstract - page 2, line 39.  More provide greater details on page 14, lines 255-262. In brief, we first inductively analyzed the qualitative data to identify emerging themes using a code book developed and iteratively refined as the analysis progressed. To integrate the qualitative and quantitative findings, the quantitative findings were mapped onto the existing themes and sub-themes from the qualitative findings, and additional themes were created where the existing themes couldn't fit.  We have reflected this analytical approach in the presentation of findings. |
| 9. Avoid ambiguity in use of phrases such as those on page 19 under divorce and separation subtheme: Some studies (how many were they? Several studies corroborated… (add number of studies if possible). | This has been addressed throughout the manuscript. See examples on page 21, line 374; page 19, line 347. |
| 10. For balanced reporting, summarize qualitative reports similar to how you report key statistics from quantitative papers. | Addressed throughout the manuscript. |
| 11. Physical and emotional abuse (Line 325-347) instead of highlighting what each study reported, you could pull together similar forms of abuse and highlight the countries represented while connecting one form of abuse to another. This can help demonstrate the authors’ level of understanding of the effects of infertility on the victims. | We appreciate this comment.  We have fused the presentation of violence into a synthesised summary. See page 19 and 20, lines 347-356. |
| 12. Pay attention to the tense used to make a distinction of your own reporting versus what the papers under review reported. For example, line 350-354, the authors interchangeably use past and present continuous tenses when highlighting what the papers under review reported. | We are apologetic for this oversight, and have now consistently used reported speech to cover every part of the results, except where we are quoting directly, and we apply the quotation marks. This was addressed. |
| 13. Theme 1 and associated subthemes are quite comprehensive. Is it possible to rearrange the subthemes and the narrative synthesis starting with those emerging early in the relationship and progressively moving towards extremes such as separation and divorce? This can help highlight events chronologically as might be expected in the natural context of infertility. | We appreciate this comment, and this has now been addressed by reordering the themes chronologically based on how they would typically occur.  We start with reduced sexual satisfaction, infidelity, physical and emotional abuse to separation and divorce- see pages 18-21. |
| 14. Theme 2: Infertility and mental health: Line 361 starts with “Several studies described a complex interplay of mental/psychological distress and infertility”. As might be expected for any interested reader, I was very eager to see the complexity of the reported relationship between infertility and mental health. The authors however went on to offer only descriptive statistics reported in individual papers. How complex is this? | We again appreciate the reviewer for highlighting this important point.  We revised by fusing the findings from the individual studies to showcase the complexity of infertility and mental health.  In brief we present the epidemiological burden of depression, anxiety, emotional distress, and declining psychological functioning among those with infertility. Distress often intensifies with prolonged infertility or repeated treatment failure, and in few cases includes suicidal ideation. Qualitative studies show the role of uncertainty, fear of the future, and unmet social expectations drive persistent worry, sadness, and emotional exhaustion. We also show the role/place of enacted/internalised stigma across family, community, workplace, and religious settings, and how that results in social exclusion |
| 15. The expected complexity of the relationship between infertility and mental health could be reported under specific subthemes. For a paper examining the effects of infertility on mental health, it is not clear how Experiences of stigma and discrimination; and coping mechanisms are effects of infertility. | We agree with the reviewer that stigma and discrimination are part of the mental health theme. We have revised and merged these themes. We however think the coping mechanism theme should be presented separately to highlight how individuals cope with the effects of infertility. |
| 16. I would suggest that Experiences of stigma and discrimination; and coping mechanisms instead of being standalone themes be used to examine the complexity of the relationship between infertility and mental health. For example, demonstrate whether infertile women and/or men facing stigma and discrimination report severe/higher levels of mental health problems (anxiety/depression etc.) compared to those who don’t report those associated issues. | We thank the reviewer for this important suggestion. We agree that stigma and discrimination can influence mental health outcomes among individuals experiencing infertility.  However, in the studies included in this scoping review, the evidence mainly described stigma and discrimination as experiences associated with infertility, without explicitly examining or quantifying their relationship with the severity or levels of mental health outcomes such as anxiety or depression. Similarly, coping mechanisms were discussed as separate responses to infertility-related challenges rather than as factors directly linked to mental health outcomes. |
| 17. Similarly, regarding coping mechanisms, be clear whether maladaptive/adaptive coping relate to experiences of infertility or effects of infertility such as mental health. What are these coping mechanisms addressing? Does your literature review show that women and/or men using adaptive coping strategies have better mental health than those utilizing maladaptive coping strategies? What qualitative experiences, if any, support such an observation? Are their differences between men and women regarding coping mechanisms? | We thank the reviewer for this insightful comment. We have revised the manuscript to clarify the conceptual distinction between what individuals are coping with and how coping relates to experiences around stigma and discrimination as well as mental health outcomes.  In particular - both the adaptive and maladaptive coping strategies were addressing the experience of infertility itself, including its social consequences and none of the coping responses targeted mental health symptoms per se, but rather at the stressors generated by infertility — such as social pressure, repeated treatment failure, and fear of relationship dissolution.  Looking at the evidence linking coping style to mental health outcomes, the literature was very shallow and these was just 1 study that suggested that adaptive coping (e.g., positive religious coping, seeking or obtaining social support) was associated with better psychological well‑being, while maladaptive coping (e.g., denial, avoidance, defensiveness) is linked to greater distress and isolation. |
| 18. NOTE: Whereas I agree that stigma and discrimination are commonly associated with infertility, I cannot say that Theme 3 (Experiences of stigma and discrimination) is part of the effects of, or outcomes of infertility, as per the aim of the current review. It might help to clearly define and stay within the boundaries of the current review. | We agree with the above comment and went ahead and merged the experiences of stigma and discrimination with mental health. |
| 19. ***Discussion:*** Similar to the background section, the authors pay a lot of attention on available or lack of interventions for effects of infertility on mental health and quality of relationship. In keeping with the aim of the review, I would have wanted to see the authors’ arguments on the implications of effects on infertility (mental health and relationship problems) on affected individuals and couples before proposing the need for interventions. | We thank the reviewer for the feedback.  We have revised the discussion section and added a paragraph discussing the implications of the effects of infertility on mental health and relationship problems among affected individuals and couples (page 31, lines 620-629 of the manuscript). |
| 20. ***Conclusion:*** this seems to be missing. Please provide a concise conclusion of your review findings under a clear heading. | We have now added a concise conclusion to the manuscript under a clear heading on page 34, lines 690-702 |

**Reviewer #2:**

| **Reviewer comment** | **Response** |
| --- | --- |
| The script was clearly written with adequate evidence showing the effects of infertility on relationship quality and mental health. However, there was a theoretical gap with the finding of the review. The author could greatly benefit from adding psychological theories that backup patient care. | Thank you for the feedback. We have added a theoretical framework to the introduction section of the manuscript (lines 128–142) |

**Editorial comments**

- Clarify the scope, target population and reporting structure of the review

This has been comprehensively addressed.

In response we have added details clarifying the review scope or topic of interest, type of studies, type of participants/target population and setting. We have also clarified the reporting structure of the review on page 9, line 159-160), including attaching as supplementary file the PRISMA-ScR checklist.

Additional details including the; review question (page 8, lines 142-144), purpose of the scoping review (page 8, lines 145-154), type of studies (page 9, lines 165-177), topic of interest (page 10, lines 182-191), participants (page 10, lines 192-195) and setting (page 11, lines 196-199).

- Refine and clarify the thematic framework to ensure in aligns with study aims in relation to infertility and relationship quality and mental health

We appreciate the editorial team for this comment. We have addressed this comment by proposing a theoretical framework to properly contextualize the study and improve the discussion and interpretation of the review findings. These details are in page 7 and 8, lines 127-141.

- Improve the presentation of the findings in the tables to reflect key study characteristics

Addressed - we have provided additional details and contents and also appropriately outlined our themes in the appendix S3, and below provided justification for this approach.

| **Reviewer comment** | **Response** |
| --- | --- |
| Figure 2: please;   a) provide a direct link to the base layer of the map (i.e., the country or region border shape) and ensure this is also included in the figure legend; | This has been provided - as an additional file with the base maps have also been uploaded. |
| a) Provide a link to the terms of use / license information for the base layer image or shapefile.   We cannot publish proprietary or copyrighted maps (e.g. Google Maps, Mapquest) and the terms of use for your map base layer must be compatible with our CC-BY 4.0 license. | This has been provided. The map layout was designed by authors from APHRC and is free for manipulation and use. All copyrights are compatible with your CC-BY 4.0 license. |

**Journal Requirements:**

1. Please provide a detailed online Financial Disclosure statement. This is published with the article. It must therefore be completed in full sentences and contain the exact wording you wish to be published.

- Provided

2. Please clarify all sources of financial support for your study. List the grants, grant numbers, and organizations that funded your study, including funding received from your institution. Please note that suppliers of material support, including research materials, should be recognized in the Acknowledgements section rather than in the Financial Disclosure.

Done

3. State the initials, alongside each funding source, of each author to receive each grant. For example: “This work was supported by the National Institutes of Health (####### to AM; ###### to CJ) and the National Science Foundation (###### to AM).”

Done

4. State what role the funders took in the study. If the funders had no role in your study, please state: “The funders had no role in study design, data collection and analysis, decision to publish, or preparation of the manuscript.”

If you did not receive any funding for this study, please simply state: “The authors received no specific funding for this work.”

For more information, please go to our submission guidelines:
<https://journals.plos.org/globalpublichealth/s/submission-guidelines#loc-financial-disclosure-statement>

Done

5. Please ensure that the funders and grant numbers match between the Financial Disclosure field and the Funding Information tab in your submission form. Note that the funders must be provided in the same order in both places as well.

**Done**

6. We note that your Data Availability Statement is currently as follows: "All data and related metadata underlying the findings reported in this manuscripts have been submitted as part of this manuscript. Additional material have already been provided as part of the submitted article supporting information."

Please confirm at this time whether or not your submission contains all raw data required to replicate the results of your study. Authors must share the “minimal data set” for their submission. PLOS defines the minimal data set to consist of the data required to replicate all study findings reported in the article, as well as related metadata and methods (<https://journals.plos.org/globalpublichealth/s/data-availability#loc-minimal-data-set-definition>).

For example, authors should submit the following data:

- The values behind the means, standard deviations and other measures reported;
- The values used to build graphs;
- The points extracted from images for analysis.

Authors do not need to submit their entire data set if only a portion of the data was used in the reported study.

If your submission does not contain these data, please either upload them as Supporting Information files or deposit them to a stable, public repository and provide us with the relevant URLs, DOIs, or accession numbers. For a list of recommended repositories, please see <https://journals.plos.org/globalpublichealth/s/recommended-repositories>.

If there are ethical or legal restrictions on sharing a de-identified data set, please explain them in detail (e.g., data contain potentially sensitive information, data are owned by a third-party organization, etc.) and who has imposed them (e.g., an ethics committee). Please also provide contact information for a data access committee, ethics committee, or other institutional body to which data requests may be sent. If data are owned by a third party, please indicate how others may request data access.

Done

7. Please provide separate main figure files in .tif or .eps format only and ensure that all files are under our size limit of 10MB.

For more information about how to convert your figure files please see our guidelines: <https://journals.plos.org/globalpublichealth/s/figures>

8. Please include a separate legend or caption for each figure in your main manuscript.
9. Some material included in your submission may be copyrighted. According to PLOS’s copyright policy, authors who use figures or other material (e.g., graphics, clipart, maps) from another author or copyright holder must demonstrate or obtain permission to publish this material under the Creative Commons Attribution 4.0 International (CC BY 4.0) License used by PLOS journals. Please closely review the details of PLOS’s copyright requirements here: PLOS Licenses and Copyright. If you need to request permissions from a copyright holder, you may use PLOS's Copyright Content Permission form.

Please respond directly to this email or email the journal office and provide any known details concerning your material's license terms and permissions required for reuse, even if you have not yet obtained copyright permissions or are unsure of your material's copyright compatibility.

Potential Copyright Issues:

**Figure 2: please**

(a) provide a direct link to the base layer of the map (i.e., the country or region border shape) and ensure this is also included in the figure legend; and (b) provide a link to the terms of use / license information for the base layer image or shapefile. We cannot publish proprietary or copyrighted maps (e.g. Google Maps, Mapquest) and the terms of use for your map base layer must be compatible with our CC-BY 4.0 license.

Note: if you created the map in a software program like R or ArcGIS, please locate and indicate the source of the basemap shapefile onto which data has been plotted.

If your map was obtained from a copyrighted source please amend the figure so that the base map used is from an openly available source. Alternatively, please provide explicit written permission from the copyright holder granting you the right to publish the material under our CC-BY 4.0 license.

Please note that the following CC BY licenses are compatible with PLOS license: CC BY 4.0, CC BY 2.0 and CC BY 3.0, meanwhile such licenses as CC BY-ND 3.0 and others are not compatible due to additional restrictions.

If you are unsure whether you can use a map or not, please do reach out and we will be able to help you. The following websites are good examples of where you can source open access or public domain maps: 
* U.S. Geological Survey (USGS) - All maps are in the public domain. (<http://www.usgs.gov>) 
* PlaniGlobe - All maps are published under a Creative Commons license so please cite “PlaniGlobe, <http://www.planiglobe.com>, CC BY 2.0” in the image credit after the caption. (<http://www.planiglobe.com/?lang=enl>) 
* Natural Earth - All maps are public domain. (<http://www.naturalearthdata.com/about/terms-of-use/>

**Done**
