# Supplementary material for: Effects of infertility on relationship quality and mental health outcomes in sub-Saharan Africa: A scoping review
Source: PLOS Glob Public Health. 2026 Nov 10;6(11):e0005923. doi: 10.1371/journal.pgph.0005923 (PMC13750579; doi:10.1371/journal.pgph.0005923)
Supplement: Supplementary file 7 [file pgph.0005923.s007.docx]

Reviewers’ Response Table Example

|  | First Review | Second Review |  |
| --- | --- | --- | --- |
| Background | The background section is comprehensive with citations that are up to date  The script is written is a clear manner with no ambiguity | Adequate |  |
|  | The problem has been clearly highlighted in the text | . |  |
| Literature review | The literature review is very detailed. However, 62% of the data is from west Africa which may not be representative for the Sub-Saharan Africa region. | The literature review is adequately representative of the Sub-Saharan African Region |  |
|  | The mental health challenges have been clearly highlighted |  |  |
| Methodology | The methodology is satisfactory. There is balance between the qualitative and quantitative peer-reviewed articles | Adequate |  |
|  | However the argument that psychological interventions should be embedded in the care of infertility lacks theoretical backing. Addition of a theory that underpins the findings could be relevant |  |  |
| Research Ethical Practices | Ethical considerations have been adequately explained |  |  |
| Overall comment | The review satisfactorily displays the effect of infertility on relationship satisfaction and mental health outcomes in SSA however there is a big theoretical gap that would strengthen the findings | The gaps highlighted in the first review have been **addressed**. |  |
