# Supplementary material for: Effects of infertility on relationship quality and mental health outcomes in sub-Saharan Africa: A scoping review
Source: PLOS Glob Public Health. 2026 Nov 10;6(11):e0005923. doi: 10.1371/journal.pgph.0005923 (PMC13750579; doi:10.1371/journal.pgph.0005923)
Supplement: Supplementary file 8 [file pgph.0005923.s008.docx]

The authors did a meticulous revision based on the editor and reviewers comments, which has substantially improved the quality of the manuscript. However, some remaining points warrant correction.

The date of the review is inconsistent and varies from one section to the other. It is 2000 to January 2026 in the abstract, 2000 to November 2025 in the review question and an update in December 2025 in the Methods.

The authors stated that “no studies or reviews” exist in the topic. However, this claim is not accurate as other studies and reviews are available in the literature. Instead, the specificity of this scoping review could be highlighted.

The authors should limit the causal relationship between variables to the experimental studies. Otherwise, the association terminologies should be used as the majority of the studies are observational.

As highlighted by reviewer 1, the authors should make the evidence related to each major theme more explicit. Appendix S3 helps, but it would be more interesting to identify the number of studies that support each theme or subtheme. This would give strength to the themes and subthemes based on the number of studies that focused on them.

It is necessary to mention in the discussion or study limitations that since the scoping review does not require a critical appraisal, the provided evidence maybe strengthened or supported by conducting in the future a systematic review about the topic.

Edit the manuscript for English and grammatical mistakes. For example, Orchid ID should be ORCID iD.
