# Supplementary material for: Effects of infertility on relationship quality and mental health outcomes in sub-Saharan Africa: A scoping review
Source: PLOS Glob Public Health. 2026 Nov 10;6(11):e0005923. doi: 10.1371/journal.pgph.0005923 (PMC13750579; doi:10.1371/journal.pgph.0005923)
Supplement: Supplementary file 9 [file pgph.0005923.s009.docx]

**PGPH-D-26-00060R1**

September 25, 2026

**Revisions: Effects of infertility on relationship quality and mental health outcomes in sub-Saharan Africa: a scoping review**

Thank you for submitting your manuscript to PLOS Global Public Health. After careful consideration, we feel that it has merit but does not fully meet PLOS Global Public Health’s publication criteria as it currently stands. Therefore, we invite you to submit a revised version of the manuscript that addresses the points raised by the editor.

**Editors' comments:**

The authors did a meticulous revision based on the editor and reviewers’ comments, which has substantially improved the quality of the manuscript. However, some remaining points warrant correction.

We thank the editor for the assessment and the opportunity to resubmit this manuscript. We addressed all editorial comments in the response letter below, with revisions tracked throughout the revised manuscript. We have submitted both the highlighted and the clean revised manuscript.

| **Reviewer comment** | **Response** |
| --- | --- |
| **Abstract** | |
| 1. The date of the review is inconsistent and varies from one section to the other. It is 2000 to January 2026 in the abstract, 2000 to November 2025 in the review question and an update in December 2025 in the Methods. | We thank the editor for pointing this inconsistency. The date of review indicated in the abstract was incorrect. We have therefore corrected it to "2000 to November 2025," consistent with the dates in the review question and Methods section.  Changes are found on page 3, line 38, in the revised manuscript. |
| ***Background***: | |
| 2. The authors stated that “no studies or reviews” exist in the topic. However, this claim is not accurate as other studies and reviews are available in the literature. Instead, the specificity of this scoping review could be highlighted | We appreciate this feedback and agree with the reviewer's point. We have revised the manuscript to restate our claim more accurately. Rather than stating that 'no studies or reviews exist,' we now emphasize that no comprehensive scoping reviews specifically examining the intersection of infertility, relationship quality, and mental health outcomes in sub-Saharan Africa have been conducted to date. These changes are made in page 7, lines 115-118. |
| **Results and discussion:** |  |
| 3. The authors should limit the causal relationship between variables to the experimental studies. Otherwise, the association terminologies should be used as the majority of the studies are observational. | Thank you for this helpful feedback. We have revised the manuscript to replace causal language with language reflecting associations, except where causal inferences are supported by experimental studies or where we have retained direct quotations from qualitative studies. Few examples of these changes can be found on page 10, line 192; page 18, line 321; page 19, line 333. |
| 4. The authors should make the evidence related to each major theme more explicit. Appendix S3 helps, but it would be more interesting to identify the number of studies that support each theme or subtheme. This would give strength to the themes and subthemes based on the number of studies that focused on them. | Thank you for this valuable suggestion. We agree that reporting the number of studies supporting each theme and subtheme could further strengthen the presentation of the findings.   - We have added the total number of studies per theme in the results section of the revised manuscript. Changes are on page 18, lines 313 and 319. Page 19, line 338; page 20, line 359; page 21, line 386. - In addition, we added a column in Appendix S3 that provides detailed information on the themes and subthemes the records/studies contribute to, allowing readers to assess the breadth of the evidence underpinning the synthesis. |
| 5. It is necessary to mention in the discussion or study limitations that since the scoping review does not require a critical appraisal, the provided evidence maybe strengthened or supported by conducting in the future a systematic review about the topic. | We have added a limitation noting that scoping reviews do not require a critical appraisal of the included studies. We incorporated this into the Limitations section of the manuscript on page 34, lines 682–686. |
| 6. Edit the manuscript for English and grammatical mistakes. For example, Orchid ID should be ORCID iD. | We thank you for pointing this out. We have thoroughly reviewed the manuscript and made all the necessary adjustments. |
