# Supplementary material for: Effects of infertility on relationship quality and mental health outcomes in sub-Saharan Africa: A scoping review
Source: PLOS Glob Public Health. 2026 Nov 10;6(11):e0005923. doi: 10.1371/journal.pgph.0005923 (PMC13750579; doi:10.1371/journal.pgph.0005923)
Supplement: Peer review history

# Author comment: Author response to Decision Letter 0

**DOI:** 10.1371/journal.pgph.0005923.r001

15 Jan 2026

Attachment Submitted filename: Response letter for PGPH.docx

# Peer review: Decision Letter 0

**DOI:** 10.1371/journal.pgph.0005923.r002

**Authors:** Margaret Mutumba

18 Mar 2026

PGPH-D-26-00060

Effects of infertility on relationship quality and mental health outcomes in sub-Saharan Africa: a scoping review

PLOS Global Public Health

Dear Dr. Juma,

Thank you for submitting your manuscript to PLOS Global Public Health. After careful consideration, we feel that it has merit but does not fully meet PLOS Global Public Health’s publication criteria as it currently stands. Therefore, we invite you to submit a revised version of the manuscript that addresses the points raised during the review process.

Be sure to:

- Clarify scope, target population and reporting structure of review
- Refine and clarify the thematic framework to ensure in aligns with study aims in relation to infertility and relationship quality and mental health
- Improve the presentation of the findings in the tables to reflect key study characteristics

Please submit your revised manuscript by Apr 17 2026 11:59PM. If you will need more time than this to complete your revisions, please reply to this message or contact the journal office at globalpubhealth@plos.org. When you're ready to submit your revision, log on to https://www.editorialmanager.com/pgph/ and select the 'Submissions Needing Revision' folder to locate your manuscript file.

Please include the following items when submitting your revised manuscript:

- A letter that responds to each point raised by the editor and reviewer(s). You should upload this letter as a separate file labeled 'Response to Reviewers'.
- A marked-up copy of your manuscript that highlights changes made to the original version. You should upload this as a separate file labeled 'Revised Manuscript with Track Changes'.
- An unmarked version of your revised paper without tracked changes. You should upload this as a separate file labeled 'Manuscript'.

Guidelines for resubmitting your figure files are available below the reviewer comments at the end of this letter.

We look forward to receiving your revised manuscript.

Kind regards,

Margaret Mutumba, Ph.D

Academic Editor

PLOS Global Public Health

Journal Requirements:

1. Please provide a detailed online Financial Disclosure statement. This is published with the article. It must therefore be completed in full sentences and contain the exact wording you wish to be published.

a) Please clarify all sources of financial support for your study. List the grants, grant numbers, and organizations that funded your study, including funding received from your institution. Please note that suppliers of material support, including research materials, should be recognized in the Acknowledgements section rather than in the Financial Disclosure.

b) State the initials, alongside each funding source, of each author to receive each grant. For example: “This work was supported by the National Institutes of Health (####### to AM; ###### to CJ) and the National Science Foundation (###### to AM).”

c) State what role the funders took in the study. If the funders had no role in your study, please state: “The funders had no role in study design, data collection and analysis, decision to publish, or preparation of the manuscript.”

If you did not receive any funding for this study, please simply state: “The authors received no specific funding for this work.”

For more information, please go to our submission guidelines:

https://journals.plos.org/globalpublichealth/s/submission-guidelines#loc-financial-disclosure-statement

2. Please ensure that the funders and grant numbers match between the Financial Disclosure field and the Funding Information tab in your submission form. Note that the funders must be provided in the same order in both places as well.

3. We note that your Data Availability Statement is currently as follows: "All data and related metadata underlying the findings reported in this manuscripts have been submitted as part of this manuscript. Additional material have already been provided as part of the submitted article supporting information."

Please confirm at this time whether or not your submission contains all raw data required to replicate the results of your study. Authors must share the “minimal data set” for their submission. PLOS defines the minimal data set to consist of the data required to replicate all study findings reported in the article, as well as related metadata and methods (https://journals.plos.org/globalpublichealth/s/data-availability#loc-minimal-data-set-definition).

For example, authors should submit the following data:

- The values behind the means, standard deviations and other measures reported;

- The values used to build graphs;

- The points extracted from images for analysis.

Authors do not need to submit their entire data set if only a portion of the data was used in the reported study.

If your submission does not contain these data, please either upload them as Supporting Information files or deposit them to a stable, public repository and provide us with the relevant URLs, DOIs, or accession numbers. For a list of recommended repositories, please see https://journals.plos.org/globalpublichealth/s/recommended-repositories.

If there are ethical or legal restrictions on sharing a de-identified data set, please explain them in detail (e.g., data contain potentially sensitive information, data are owned by a third-party organization, etc.) and who has imposed them (e.g., an ethics committee). Please also provide contact information for a data access committee, ethics committee, or other institutional body to which data requests may be sent. If data are owned by a third party, please indicate how others may request data access.

4. Please provide separate main figure files in .tif or .eps format only and ensure that all files are under our size limit of 10MB.

For more information about how to convert your figure files please see our guidelines: https://journals.plos.org/globalpublichealth/s/figures

5. Please include a separate legend or caption for each figure in your main manuscript.

6. Some material included in your submission may be copyrighted. According to PLOS’s copyright policy, authors who use figures or other material (e.g., graphics, clipart, maps) from another author or copyright holder must demonstrate or obtain permission to publish this material under the Creative Commons Attribution 4.0 International (CC BY 4.0) License used by PLOS journals. Please closely review the details of PLOS’s copyright requirements here: PLOS Licenses and Copyright. If you need to request permissions from a copyright holder, you may use PLOS's Copyright Content Permission form.

Please respond directly to this email or email the journal office and provide any known details concerning your material's license terms and permissions required for reuse, even if you have not yet obtained copyright permissions or are unsure of your material's copyright compatibility.

Potential Copyright Issues:

Figure 2: please (a) provide a direct link to the base layer of the map (i.e., the country or region border shape) and ensure this is also included in the figure legend; and (b) provide a link to the terms of use / license information for the base layer image or shapefile. We cannot publish proprietary or copyrighted maps (e.g. Google Maps, Mapquest) and the terms of use for your map base layer must be compatible with our CC-BY 4.0 license.

Note: if you created the map in a software program like R or ArcGIS, please locate and indicate the source of the basemap shapefile onto which data has been plotted.

If your map was obtained from a copyrighted source please amend the figure so that the base map used is from an openly available source. Alternatively, please provide explicit written permission from the copyright holder granting you the right to publish the material under our CC-BY 4.0 license.

Please note that the following CC BY licenses are compatible with PLOS license: CC BY 4.0, CC BY 2.0 and CC BY 3.0, meanwhile such licenses as CC BY-ND 3.0 and others are not compatible due to additional restrictions.

If you are unsure whether you can use a map or not, please do reach out and we will be able to help you. The following websites are good examples of where you can source open access or public domain maps:

* U.S. Geological Survey (USGS) - All maps are in the public domain. (http://www.usgs.gov)

* PlaniGlobe - All maps are published under a Creative Commons license so please cite “PlaniGlobe, http://www.planiglobe.com, CC BY 2.0” in the image credit after the caption. (http://www.planiglobe.com/?lang=enl)

* Natural Earth - All maps are public domain. (http://www.naturalearthdata.com/about/terms-of-use/)

7. If the reviewer comments include a recommendation to cite specific previously published works, please review and evaluate these publications to determine whether they are relevant and should be cited. There is no requirement to cite these works unless the editor has indicated otherwise.

8. Please review your reference list to ensure that it is complete and correct. If you have cited papers that have been retracted, please include the rationale for doing so in the manuscript text, or remove these references and replace them with relevant current references. Any changes to the reference list should be mentioned in the rebuttal letter that accompanies your revised manuscript. If you need to cite a retracted article, indicate the article’s retracted status in the References list and also include a citation and full reference for the retraction notice.

Additional Editor Comments (if provided):

[Note: HTML markup is below. Please do not edit.]

Reviewers' comments:

Reviewer's Responses to Questions

**Comments to the Author**

1. Does this manuscript meet PLOS Global Public Health’s publication criteria?

Reviewer #1: Partly

Reviewer #2: Yes

**********

2. Has the statistical analysis been performed appropriately and rigorously?

Reviewer #1: N/A

Reviewer #2: Yes

**********

3. Have the authors made all data underlying the findings in their manuscript fully available (please refer to the Data Availability Statement at the start of the manuscript PDF file)?

The PLOS Data policy

Reviewer #1: No

Reviewer #2: Yes

**********

4. Is the manuscript presented in an intelligible fashion and written in standard English?

Reviewer #1: Yes

Reviewer #2: Yes

**********

Reviewer #1: Manuscript Title: Effects of infertility on relationship quality and mental health outcomes in sub-Saharan Africa: a scoping review

I appreciate the authors for undertaking this work, and the editor for the opportunity to review this manuscript on a very important topic of effects of infertility in sub-Saharan Africa. Below are my observations and comments.

Abstract

1. Include the target population in the abstract to help the reader start off with the right perspective.

Background

2. The aim of this scoping review was to examine the effects of infertility on relationship quality and mental health outcomes in SSA. It would help to specify the target population for the primary studies reviewed.

3. The search strategy is satisfactorily elaborated.

Methods

4. Assessment of methodological quality reported as not done. Please add citation supporting this position, if any.

Results

Figure 1 (PRISMA flow diagram).

5. Be explicit on the reasons for exclusion of papers, either within the diagram or the narrative that follows, with specific number of papers excluded at each stage.

6. Table 3 showing the characteristics of the study included can be enhanced by, combining some aspects in same row (e.g. Author, country, aim of the study) and adding important information such as study design, measures used and key findings. This would add transparency and ensure credibility of the results reported. A table in landscape would accommodate key information on the same page. Key information for each study in the same row would enhance accessibility for the reader. For example, a reader would see which study had only females, males or both at a glance.

7. Key themes: As noted above, it would help to first summarize the key findings per paper in table 3 before showing the themes that emerge from the review. In its current form we don’t know for example how many papers reviewed reported on aspects of infertility and relationship quality and it therefore remains unclear how this theme emerged from your review.

8. A descriptive account of the results from each paper/country rather than a clear synthesis of similar results is given. Since the review includes papers employing diverse study designs, it would help to see how the authors try to integrate findings from different designs.

9. Avoid ambiguity in use of phrases such as those on page 19 under divorce and separation subtheme: Some studies (how many were they? Several studies corroborated… (add number of studies if possible).

10. For balanced reporting, summarize qualitative reports similar to how you report key statistics from quantitative papers.

11. Physical and emotional abuse (Line 325-347) instead of highlighting what each study reported, you could pull together similar forms of abuse and highlight the countries represented while connecting one form of abuse to another. This can help demonstrate the authors’ level of understanding of the effects of infertility on the victims.

12. Pay attention to the tense used to make a distinction of your own reporting versus what the papers under review reported. For example, line 350-354, the authors interchangeably use past and present continuous tenses when highlighting what the papers under review reported.

13. Theme 1 and associated subthemes are quite comprehensive. Is it possible to rearrange the subthemes and the narrative synthesis starting with those emerging early in the relationship and progressively moving towards extremes such as separation and divorce? This can help highlight events chronologically as might be expected in the natural context of infertility.

14. Theme 2: Infertility and mental health: Line 361 starts with “Several studies described a complex interplay of mental/psychological distress and infertility”. As might be expected for any interested reader, I was very eager to see the complexity of the reported relationship between infertility and mental health. The authors however went on to offer only descriptive statistics reported in individual papers. How complex is this?

15. The expected complexity of the relationship between infertility and mental health could be reported under specific subthemes. For a paper examining the effects of infertility on mental health, it is not clear how Experiences of stigma and discrimination; and coping mechanisms are effects of infertility.

16. I would suggest that Experiences of stigma and discrimination; and coping mechanisms instead of being standalone themes be used to examine the complexity of the relationship between infertility and mental health. For example, demonstrate whether infertile women and/or men facing stigma and discrimination report severe/higher levels of mental health problems (anxiety/depression etc.) compared to those who don’t report those associated issues.

17. Similarly, regarding coping mechanisms, be clear whether maladaptive/adaptive coping relate to experiences of infertility or effects of infertility such as mental health. What are these coping mechanisms addressing? Does your literature review show that women and/or men using adaptive coping strategies have better mental health than those utilizing maladaptive coping strategies? What qualitative experiences, if any, support such an observation? Are their differences between men and women regarding coping mechanisms?

18. NOTE: Whereas I agree that stigma and discrimination are commonly associated with infertility, I cannot say that Theme 3 (Experiences of stigma and discrimination) is part of the effects of, or outcomes of infertility, as per the aim of the current review. It might help to clearly define and stay within the boundaries of the current review.

19. Discussion: Similar to the background section, the authors pay a lot of attention on available or lack of interventions for effects of infertility on mental health and quality of relationship. In keeping with the aim of the review, I would have wanted to see the authors’ arguments on the implications of effects on infertility (mental health and relationship problems) on affected individuals and couples before proposing the need for interventions.

20. Conclusion: this seems to be missing. Please provide a concise conclusion of your review findings under a clear heading.

Reviewer #2: The script was clearly written with adequate evidence showing the effects of infertility on relationship quality and mental health . However, there was a theoretical gap with the finding of the review. The author could greatly benefit from adding psychological theories that backup patient care.

**********

what does this mean?). If published, this will include your full peer review and any attached files.

**Do you want your identity to be public for this peer review?**  If you choose “no”, your identity will remain anonymous but your review may still be made public.

For information about this choice, including consent withdrawal, please see our Privacy Policy

Reviewer #1: **Yes:**  Samuel Ouma

Reviewer #2: No

**********

[NOTE: If reviewer comments were submitted as an attachment file, they will be attached to this email and accessible via the submission site. Please log into your account, locate the manuscript record, and check for the action link "View Attachments". If this link does not appear, there are no attachment files.]

Attachment Submitted filename: Reviewers_Response PLOS.docx

# Author comment: Author response to Decision Letter 1

**DOI:** 10.1371/journal.pgph.0005923.r003

1 Jun 2026

Attachment Submitted filename: Response to Reviewers_May 31 2026.docx

# Peer review: Decision Letter 1

**DOI:** 10.1371/journal.pgph.0005923.r004

**Authors:** Mathilde Azar

16 Aug 2026

PGPH-D-26-00060R1

Effects of infertility on relationship quality and mental health outcomes in sub-Saharan Africa: a scoping review

PLOS Global Public Health

Dear Dr. Juma,

Thank you for submitting your manuscript to PLOS Global Public Health. After careful consideration, we feel that it has merit but does not fully meet PLOS Global Public Health’s publication criteria as it currently stands. Therefore, we invite you to submit a revised version of the manuscript that addresses the points raised during the review process.

Please submit your revised manuscript by Sep 15 2026 11:59PM. If you will need more time than this to complete your revisions, please reply to this message or contact the journal office at globalpubhealth@plos.org. When you're ready to submit your revision, log on to https://www.editorialmanager.com/pgph/ and select the 'Submissions Needing Revision' folder to locate your manuscript file.

Please include the following items when submitting your revised manuscript:

- A letter that responds to each point raised by the editor and reviewer(s). You should upload this letter as a separate file labeled 'Response to Reviewers'.
- A marked-up copy of your manuscript that highlights changes made to the original version. You should upload this as a separate file labeled 'Revised Manuscript with Track Changes'.
- An unmarked version of your revised paper without tracked changes. You should upload this as a separate file labeled 'Manuscript'.

Guidelines for resubmitting your figure files are available below the reviewer comments at the end of this letter.

As the corresponding author, your ORCID iD is verified in the submission system and will appear in the published article. PLOS supports the use of ORCID, and we encourage all coauthors to register for an ORCID iD and use it as well. Please encourage your coauthors to verify their ORCID iD within the submission system before final acceptance, as unverified ORCID iDs will not appear in the published article. *Only*  the individual author can complete the verification step; PLOS staff *cannot*  verify ORCID iDs on behalf of authors.

We look forward to receiving your revised manuscript.

Kind regards,

Mathilde Azar, PhD

Academic Editor

PLOS Global Public Health

**Journal Requirements:**

1. Please provide separate figure files in .tif or .eps format.

For more information about figure files please see our guidelines:  https://journals.plos.org/globalpublichealth/s/figures

https://journals.plos.org/globalpublichealth/s/figures#loc-file-requirements

If the reviewer comments include a recommendation to cite specific previously published works, please review and evaluate these publications to determine whether they are relevant and should be cited. There is no requirement to cite these works unless the editor has indicated otherwise.

Please review your reference list to ensure that it is complete and correct. If you have cited papers that have been retracted, please include the rationale for doing so in the manuscript text, or remove these references and replace them with relevant current references. Any changes to the reference list should be mentioned in the rebuttal letter that accompanies your revised manuscript. If you need to cite a retracted article, indicate the article’s retracted status in the References list and also include a citation and full reference for the retraction notice.

**Additional Editor Comments (if provided):**

The authors did a meticulous revision based on the editor and reviewers comments, which has substantially improved the quality of the manuscript. However, some remaining points warrant correction.

The date of the review is inconsistent and varies from one section to the other. It is 2000 to January 2026 in the abstract, 2000 to November 2025 in the review question and an update in December 2025 in the Methods.

The authors stated that “no studies or reviews” exist in the topic. However, this claim is not accurate as other studies and reviews are available in the literature. Instead, the specificity of this scoping review could be highlighted.

The authors should limit the causal relationship between variables to the experimental studies. Otherwise, the association terminologies should be used as the majority of the studies are observational.

As highlighted by reviewer 1, the authors should make the evidence related to each major theme more explicit. Appendix S3 helps, but it would be more interesting to identify the number of studies that support each theme or subtheme. This would give strength to the themes and subthemes based on the number of studies that focused on them.

It is necessary to mention in the discussion or study limitations that since the scoping review does not require a critical appraisal, the provided evidence maybe strengthened or supported by conducting in the future a systematic review about the topic.

Edit the manuscript for English and grammatical mistakes. For example, Orchid ID should be ORCID iD.

[Note: HTML markup is below. Please do not edit.]

Reviewers' comments:

Reviewer's Responses to Questions

**Comments to the Author**

Reviewer #2: All comments have been addressed

**********

publication criteria?

Reviewer #2: Yes

**********

3. Has the statistical analysis been performed appropriately and rigorously?

Reviewer #2: Yes

**********

4. Have the authors made all data underlying the findings in their manuscript fully available (please refer to the Data Availability Statement at the start of the manuscript PDF file)?

The PLOS Data policy

Reviewer #2: Yes

**********

5. Is the manuscript presented in an intelligible fashion and written in standard English?

Reviewer #2: Yes

**********

Reviewer #2: Review was uploaded with comments

**********

what does this mean?). If published, this will include your full peer review and any attached files.

**Do you want your identity to be public for this peer review?**  If you choose “no”, your identity will remain anonymous but your review may still be made public.

For information about this choice, including consent withdrawal, please see our Privacy Policy

Reviewer #2: No

**********

[NOTE: If reviewer comments were submitted as an attachment file, they will be attached to this email and accessible via the submission site. Please log into your account, locate the manuscript record, and check for the action link "View Attachments". If this link does not appear, there are no attachment files.]

**Figure Resubmissions:**

Attachment Submitted filename: Reviewers_Response_PLOS_declet_1

Attachment Submitted filename: Editor_Comments_PGPH-D-26-00060_R1.docx

# Author comment: Author response to Decision Letter 2

**DOI:** 10.1371/journal.pgph.0005923.r005

1 Oct 2026

Attachment Submitted filename: Response to Editors comments Sept 30 2026.docx

# Editor report: Decision Letter 2

**DOI:** 10.1371/journal.pgph.0005923.r006

**Authors:** Mathilde Azar

5 Oct 2026

Effects of infertility on relationship quality and mental health outcomes in sub-Saharan Africa: a scoping review

PGPH-D-26-00060R2

Dear Mr Juma,

We are pleased to inform you that your manuscript 'Effects of infertility on relationship quality and mental health outcomes in sub-Saharan Africa: a scoping review' has been provisionally accepted for publication in PLOS Global Public Health.

Before your manuscript can be formally accepted you will need to complete some formatting changes, which you will receive in a follow up email. A member of our team will be in touch with a set of requests.

Please note that your manuscript will not be scheduled for publication until you have made the required changes, so a swift response is appreciated.

IMPORTANT: The editorial review process is now complete. PLOS will only permit corrections to spelling, formatting or significant scientific errors from this point onwards. Requests for major changes, or any which affect the scientific understanding of your work, will cause delays to the publication date of your manuscript.

If your institution or institutions have a press office, please notify them about your upcoming paper to help maximize its impact. If they'll be preparing press materials, please inform our press team as soon as possible -- no later than 48 hours after receiving the formal acceptance. Your manuscript will remain under strict press embargo until 2 pm Eastern Time on the date of publication. For more information, please contact globalpubhealth@plos.org.

Thank you again for supporting Open Access publishing; we are looking forward to publishing your work in PLOS Global Public Health.

Best regards,

Mathilde Azar, PhD

Academic Editor

PLOS Global Public Health

***********************************************************

No further comments.

Reviewer Comments (if any, and for reference):
